# Subset- and tissue-defined STAT5 thresholds control homeostasis and function of innate lymphoid cells

**DOI:** 10.1084/jem.20150907

**Published:** 2017-10-02

**Authors:** Alejandro V. Villarino, Giuseppe Sciumè, Fred P. Davis, Shigeru Iwata, Beatrice Zitti, Gertraud W. Robinson, Lothar Hennighausen, Yuka Kanno, John J. O’Shea

**Affiliations:** 1 Molecular Immunology and Inflammation Branch, National Institute of Arthritis, Musculoskeletal and Skin Diseases, National Institutes of Health, Bethesda, MD; 2 Laboratory of Genetics and Physiology, National Institute of Diabetes, Digestive and Kidney Diseases, National Institutes of Health, Bethesda, MD

## Abstract

Innate lymphoid cells (ILCs) patrol environmental interfaces to defend against infection and protect barrier integrity. Using a genetic tuning model, we demonstrate that the signal-dependent transcription factor (TF) STAT5 is critical for accumulation of all known ILC subsets in mice and reveal a hierarchy of STAT5 dependency for populating lymphoid and nonlymphoid tissues. We apply transcriptome and genomic distribution analyses to define a STAT5 gene signature in natural killer (NK) cells, the prototypical ILC subset, and provide a systems-based molecular rationale for its key functions downstream of IL-15. We also uncover surprising features of STAT5 behavior, most notably the wholesale redistribution that occurs when NK cells shift from tonic signaling to acute cytokine-driven signaling, and genome-wide coordination with T-bet, another key TF in ILC biology. Collectively, our data position STAT5 as a central node in the TF network that instructs ILC development, homeostasis, and function and provide mechanistic insights on how it works at cellular and molecular levels.

## Abstract (precis)

Villarino et al. demonstrate that STAT5 is required for accumulation and function of all ILC subsets in mice. They also define a STAT5-driven transcriptional signature in NK cells and reveal a cooperative relationship with T-bet, another key ILC transcription factor.

## Introduction

Innate lymphoid cells (ILCs) patrol epithelial barriers like the skin, lungs, and intestine. They provide frontline defense against infection and tissue injury but also contribute to pathogenic inflammation and thus are viewed as key players in both protective and deleterious immune responses. A growing number of specialized ILC subsets have been codified on the basis of functional capabilities and stereotypical patterns of cytokine production and transcription factor (TF) use (Diefenbach et al., 2014; McKenzie et al., 2014; Artis and Spits, 2015). NK cells were the first to be recognized and are characterized by cytolytic activity, IFN-γ production, and the T-box family TF EOMES. Type 1 ILCs (ILC1) also produce IFN-γ but, unlike NK cells, are typically not cytolytic and do not express EOMES. Instead, they are specified by a different T-box family member, T-BET, that is also expressed by NK cells but not strictly required for their cell development (Spits et al., 2016). Type 2 ILCs (ILC2) are characterized by production of IL-5 and IL-13 and are dependent on GATA-3, along with the retinoid-related orphan receptor (ROR) family TF RORα. Type 3 ILCs (ILC3) are a heterogeneous group unified by a shared requirement for another ROR family member, RORγt. They include lymphoid tissue inducer (LTi) cells that produce both IL-17 and IL-22 and seed lymphoid organs and natural cytotoxicity receptor (NCR) 1–expressing ILC3 that produce IL-22 but do not participate in organogenesis. Like NK cells and ILC1, NCR1^+^ ILC3 express T-BET and are diminished in T-BET–deficient mice, suggesting an ontological relationship and/or lineage plasticity (Sciumé et al., 2012; Klose et al., 2013; Rankin et al., 2013).

Although each ILC subset is commonly associated with one or two lineage-defining TFs (LDTFs), a simple one-to-one instructive model fails to explain the complexity of ILC lineage specification. Instead, this process appears to be governed by multifactorial networks with overlapping nodes. Accordingly, genetic ablation of GATA-3 affects all ILC subsets, not just ILC2 (Serafini et al., 2014; Yagi et al., 2014), and there is a growing list of “multilineage” TFs (MLTFs), including ID2, NFIL3, and PLZF, that are required for the development of multiple subsets (Constantinides et al., 2014; Seillet et al., 2014; Yu et al., 2014; Xu et al., 2015). These operate in concert with LDTFs and signal-dependent TFs, such as aryl hydrocarbon receptor and NOTCH receptors, which integrate environmental or tissue-derived cues, to orchestrate a stepwise differentiation program whereby common lymphoid progenitors (CLPs) give rise to a series of ILC progenitors that sequentially lose multipotency and, ultimately, beget lineage-committed precursors for each subset (Diefenbach et al., 2014; Shih et al., 2014; De Obaldia and Bhandoola, 2015; Zook and Kee, 2016).

As with adaptive lymphocytes, ILC development and/or homeostasis is dependent on the common γ chain (γc) cytokine receptor and its dedicated tyrosine kinase, JAK3 (Vonarbourg and Diefenbach, 2012; Serafini et al., 2015; Vély et al., 2016). Consequently, ILC subsets can be categorized on the basis of their preferred γc cytokines and coreceptors; NK cells and ILC1 require IL-15 and IL-2Rβ, a component of the IL-15 receptor, whereas ILC2 and ILC3 require IL-7 and IL-7R. Because all γc cytokines deploy STAT5 as a downstream signal-dependent TF, it is also presumed to be critical for ILCs. However, until the present work, this notion had been validated only for NK cells. It has long been known that genetic ablation of STAT5 results in a profound lack of NK cells, but although this dense phenotype conveys vital importance, it precludes most functional inquiries (Moriggl et al., 1999; Yao et al., 2006; Eckelhart et al., 2011). Studies have shown that NK cell proliferation and cytotoxicity are reduced in the absence of *Stat5b*, one of the two mammalian STAT5 paralogs, but these preceded current understanding of MLTFs and LDTFs, and the underlying molecular mechanisms were not explored (Imada et al., 1998). Transcriptome-wide effects of γc cytokines have also been investigated in NK cells (Wang et al., 2015; Gotthardt et al., 2016), but these cannot be categorically attributed to STAT5, because γc cytokines also engage parallel signaling pathways, including mitogen-activated protein kinases and mTOR, that must be considered (Huntington et al., 2007; Marçais et al., 2014; Mao et al., 2016). In fact, beyond a few emblematic genes, such as those encoding BCL family proteins (Sathe et al., 2014; Shenoy et al., 2014), direct transcriptional targets of STAT5 have yet to be cataloged in this or any other ILC subset. Thus, the importance of STAT5 for NK cells is not in question but its downstream cellular and molecular consequences are loosely defined, and its impact on other ILC subsets remains to be determined.

To address these outstanding issues, we used a mouse model where STAT5 is reduced but not ablated, thereby avoiding the extreme lymphopenia, anemia, and inflammatory disease associated with complete STAT5 deficiency. Using this approach, we demonstrate that STAT5 promotes accumulation and function of all ILC subsets, as well as various types of innate-like T cells (ILTCs), and that the degree of STAT5 dependency varies according to lineage and tissue of residence. We also applied transcriptome and genomic distribution analyses to define a robust STAT5-driven transcriptional signature in NK cells, the prototypical ILC subset, and reveal pervasive effects on cellular homeostasis, lineage specification, and maturation, as well as striking differences between tonic and acute cytokine-driven STAT5 signaling and genome-wide coordination between STAT5 and T-BET, an LDTF that is common to the ILC subsets most affected by STAT5 deficiency. Collectively, our data position STAT5 as a key MLTF involved in ILC development, homeostasis, and function.

## Results

### Distinct STAT5 thresholds among ILC subsets and precursors

In mammals, STAT5 is encoded by two adjacent genes, *Stat5a* and *Stat5b*. To avoid the confounding lymphopenia, anemia, and inflammatory disease associated with ablation of both paralogs, we generated a series of mice with decreasing numbers of STAT5 alleles, ranging from four (i.e., two copies each of *Stat5a* and *Stat5b*) to one (i.e., one copy of either *Stat5a* or *Stat5b*; Villarino et al., 2016; Fig. S1 A). Strikingly, the frequency of splenic NK cells mirrored the total number of STAT5 alleles; it was slightly reduced in mice with three alleles, lower in those with two, and lower still in those with one (Fig. S1, B and C). We also noted that deletion of *Stat5b* alleles had a greater impact than deletion of *Stat5a* alleles, consistent with previous work (Imada et al., 1998).

**Figure 1.**
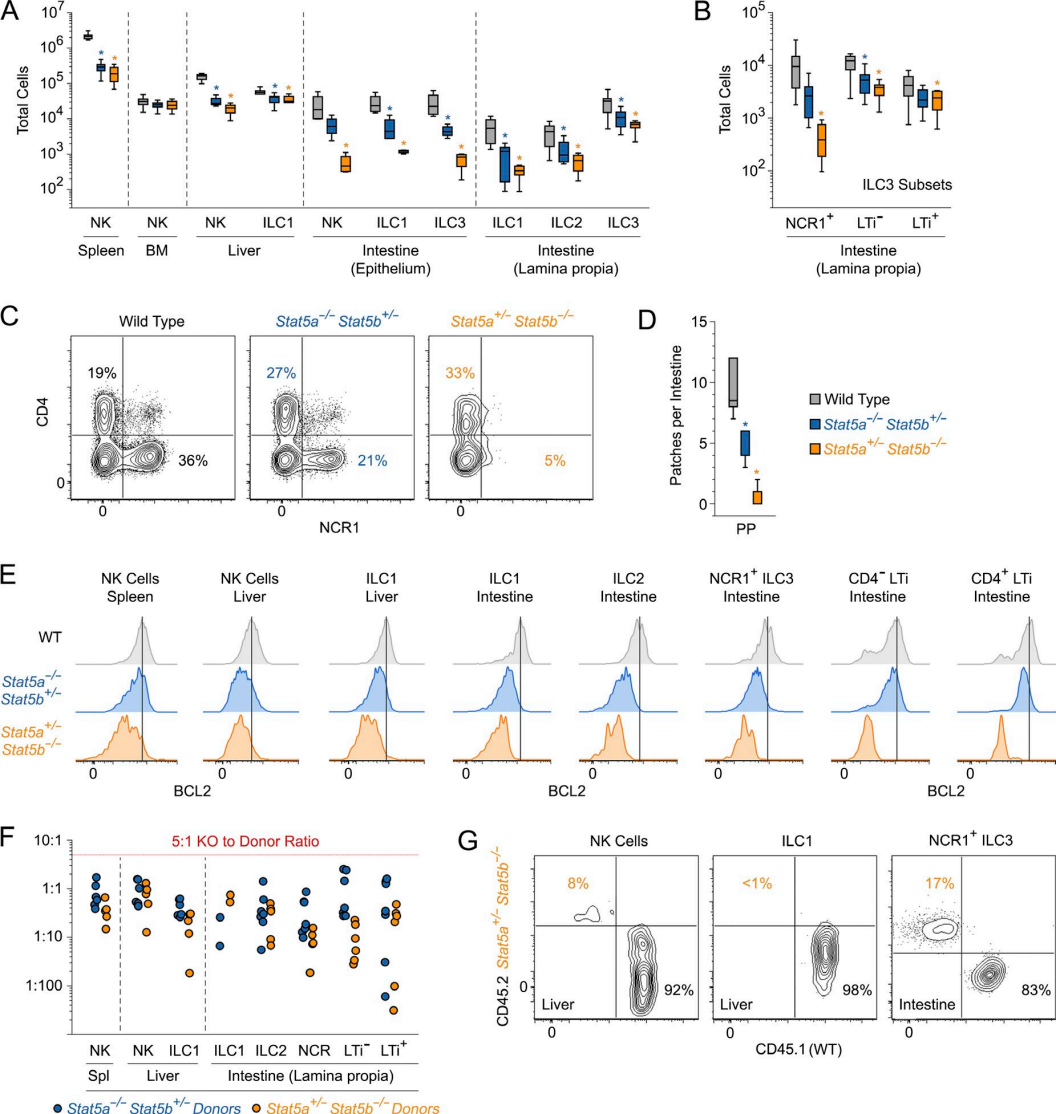
**STAT5 drives accumulation of ILCs at lymphoid and nonlymphoid tissues.** (A) Box plot shows total counts for ILC subsets within the indicated tissues. Lamina propia ILC3 refers to all IL-7R^+^ RORγt^+^ subsets. Intestinal epithelium ILC3 refers only to the NCR1^+^ subset. (B) Box plot shows total counts for ILC3 subsets in the intestinal lamina propia. NCR1^+^, NCR1^+^ ILC3; LTi^−^, CD4^−^ LTi; LTi^+^, CD4^+^ LTi. (C) Flow cytometry contour plots show percentages of CD4^+^ LTi (top left) and NCR1^+^ ILC3 (bottom right) in lamina propia. (D) Box plot shows total number of macroscopic Peyer’s patches per small intestine. (E) Flow cytometry histograms show BCL2 protein levels within the indicated subsets and tissues. (A–E) Data are compiled from or representative of three to eight experiments, depending on tissue and subset (*n* ≥ 3 mice/genotype). *, P < 0.05 (Student’s *t* test) compared with WT controls. (F) Scatterplot shows ILC engraftment after adoptive transfer of mixed bone marrow (STAT5-KO–to-WT starting ratio 5:1) into lymphopenic hosts. Each point represents an individual donor–host pair. Y axis indicates the observed STAT5-KO to WT ratio for the indicated subsets and tissues. (G) Flow cytometry contour plots show representative examples of ILC engraftment in mixed bone marrow chimeras. (F and G) Five or six donor–host pairs were assessed per genotype across two experiments. (A–G) See Fig. S2 for detailed flow cytometry gating strategies.

Contraction of splenic NK cells was most dramatic in mice bearing only one STAT5 allele, hereafter referred to as one-allele STAT5A- or STAT5B-deficient mice. Thus, we focused on these for subsequent experiments. First, we quantified NK cells in various tissues (Fig. S2, A–E). Compared with WT counterparts, one-allele STAT5A- or STAT5B-deficient mice exhibited marked reductions in the spleen, liver, and intestinal epithelium but not in the bone marrow, suggesting that migration, proliferation and/or survival defects underlie the phenotypes at peripheral sites (Fig. 1 A and Fig. S1 D). ILC1 were also diminished in the liver and intestinal epithelium (Fig. 1 A and Fig. S2, C–E), leading us to examine the intestinal lamina propia, a tissue known to be enriched for all ILC subsets (Fig. S2 F). ILC1 were, again, reduced in one-allele STAT5A- or STAT5B-deficient mice, as were GATA-3^+^ ILC2 and RORγt^+^ ILC3 (Fig. 1 A). Surprisingly, the three constituents of the ILC3 compartment were not equally affected. NCR1^+^ ILC3 were dramatically reduced, whereas CD4^−^ and CD4^+^ LTi cells were not (Fig. 1, B and C), although an attendant lack of Peyer’s patches did suggest a functional impairment (Fig. 1 D). Like their lamina propia counterparts, epithelial NCR1^+^ ILC3 were also diminished in one-allele STAT5A- or STAT5B-deficient mice (Fig. 1 A). Collectively, these data establish that STAT5 is necessary for accumulation of all ILC subsets, a conclusion strongly supported by the finding that BCL2, a known STAT5 target gene that is critical for NK cell homeostasis (Viant et al., 2017), was universally depressed (Fig. 1 E). They also make clear that STAT5B is the dominant paralog in all ILC subsets and reveal a lineage- and tissue-defined hierarchy of STAT5 dependency; some populations are clearly better able to withstand STAT5 depletion than others (e.g., liver ILC1 vs. intestinal ILC1).

Similar to ILCs, ILTCs often reside at barrier surfaces and do not require antigen-driven differentiation to execute effector functions (Cheroutre et al., 2011). In line with prior studies implicating STAT5, Jak3, γc, and associated cytokines/receptors (Cao et al., 1995; DiSanto et al., 1995; Park et al., 1995; He and Malek, 1996; Maki et al., 1996; Boesteanu et al., 1997; Suzuki et al., 1997; Lodolce et al., 1998; Kennedy et al., 2000; Ranson et al., 2003; Hoelbl et al., 2006; Klose et al., 2014a; Ettersperger et al., 2016), we found that invariant NKT cells in the spleen and liver and γδ T cells, CD4/CD8 “double-negative” T cells, and CD8αα T cells in the intestinal epithelium were diminished in one-allele STAT5A- or STAT5B-deficient mice (Fig. S1 E and Fig. S2, E and G). We also noted that, as with ILCs, deletion of STAT5B had a greater impact than deletion of STAT5A (Fig. S1 E). Thus, STAT5 is necessary for development and/or homeostasis of both ILCs and ILTCs, key lymphoid components of innate immunity.

To confirm that the ILC defects seen in one-allele STAT5A- or STAT5B-deficient mice are cell intrinsic, we transplanted a mix of WT and KO bone marrow progenitors into lymphopenic hosts and compared engraftment in the spleen, liver, and intestinal lamina propia. Despite starting with a fivefold excess of KO donor cells, WT-derived NK cells, ILC1, ILC2, and ILC3 far outpaced their KO counterparts, and, echoing the distinction seen in the donor mice, one-allele STAT5B-deficient cells were less likely to engraft than one-allele STAT5A-deficient cells (Fig. 1. F and G). Surprisingly, we found that LTi cells were rarely derived from KO donors, indicating that, despite only modest reductions in the donor mice, this subset cannot overcome STAT5 deficiency in a competitive setting (Fig. 1 F). Thus, a cell intrinsic requirement for STAT5 is evident across all ILC subsets and in multiple tissues.

**Figure 2.**
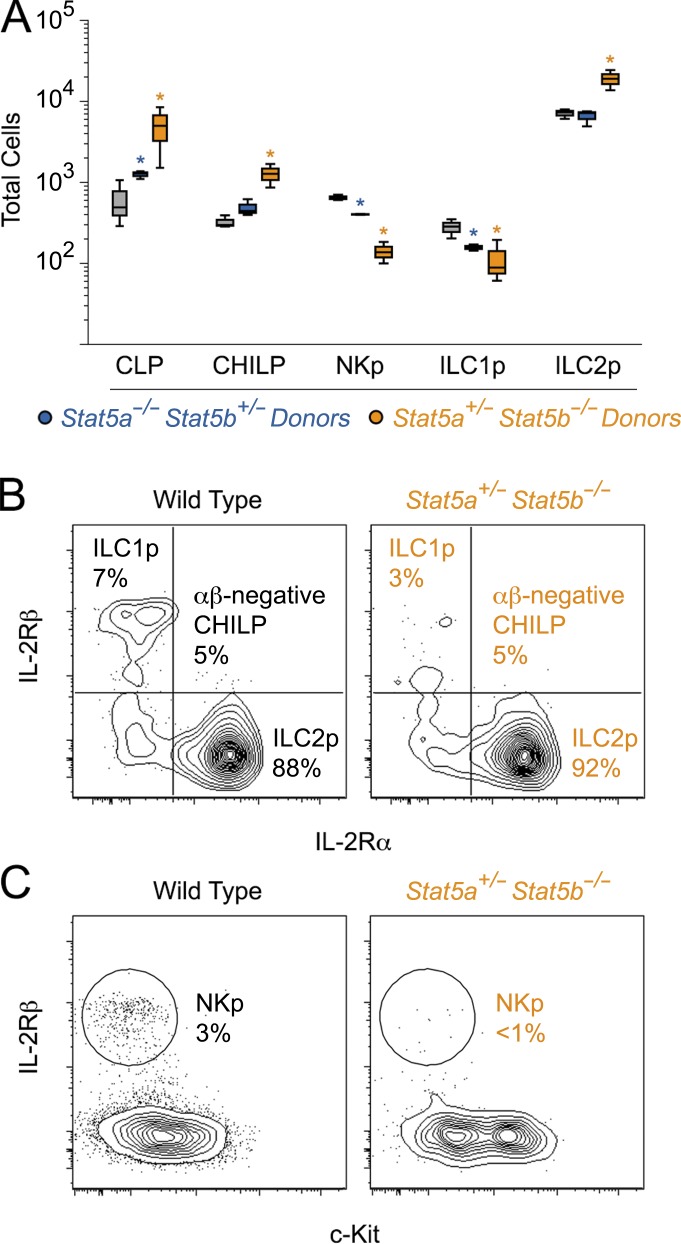
**NK and ILC1 progenitors are diminished in STAT5-deficient mice.** (A) Box plot shows total counts for common lymphoid progenitors (CLPs), common progenitors to all helper-like ILCs (CHILPs), NK cell progenitors (NKp), ILC1 progenitors (ILC1p) and ILC2 progenitors (ILC2p) in the bone marrow (BM). (B) Flow cytometry contour plots show percentages of ILC1p (top left), CHILPs (bottom left), and ILC2p (bottom right) in BM of WT or one-allele STAT5B-deficient mice. (C) Flow cytometry contour plots show percentages of NKp in BM. (A–C) Data are compiled from or representative of three experiments (*n* ≥ 3 mice/genotype). *, P < 0.05 (Student’s *t* test) compared with WT controls. See Fig. S2 for detailed flow cytometry gating strategies.

Recent work identified a common progenitor to all helper-like ILCs (CHILP) capable of generating ILC1, ILC2, or ILC3 but not NK cells, which are derived from a dedicated NK progenitor (NKp) that branches off at an earlier developmental step (Klose et al., 2014b; Yu et al., 2014). Unipotent ILC1 and ILC2 progenitors have also been described, but an ILC3 equivalent remains at large (Hoyler et al., 2012; Constantinides et al., 2015). To determine if the ILC defects seen in one-allele STAT5A- and STAT5B-deficient mice are due to an early developmental block, we quantified CLPs, which can give rise to all lymphoid lineages, CHILPs and lineage-committed NK cell, ILC1 and ILC2 progenitors (Fig. S2 H). Both CLPs and CHILPs were readily detected in one-allele STAT5A- and STAT5B-deficient mice, and in fact, both were enriched in the latter genotype (Fig. 2, A and B). A similar trend was observed for the ILC2 progenitors, suggesting that the loss of this subset in the intestine is due to defects in later development, tissue homing, and/or peripheral homeostasis (Fig. 2, A and B). In contrast, NK progenitor (NKp) and ILC1p were each diminished in one-allele STAT5B-deficient mice (less so in one-allele STAT5A-deficient mice), and the residual cells had reduced expression of IL-2Rβ (Fig. 2, A–C). Thus, beyond postdevelopmental defects, accumulation of NK cells and ILC1 at peripheral sites may be hampered by defects in the differentiation and/or fitness of their bone marrow progenitors. Considering that multipotent progenitors appear largely resistant to STAT5 depletion, we can further conclude that its influence becomes most apparent at the lineage-committed precursor stage for these two subsets and at a later developmental stage for ILC2.

### Impact of STAT5 depletion on ILC TFs and cytokines

**Figure 3.**
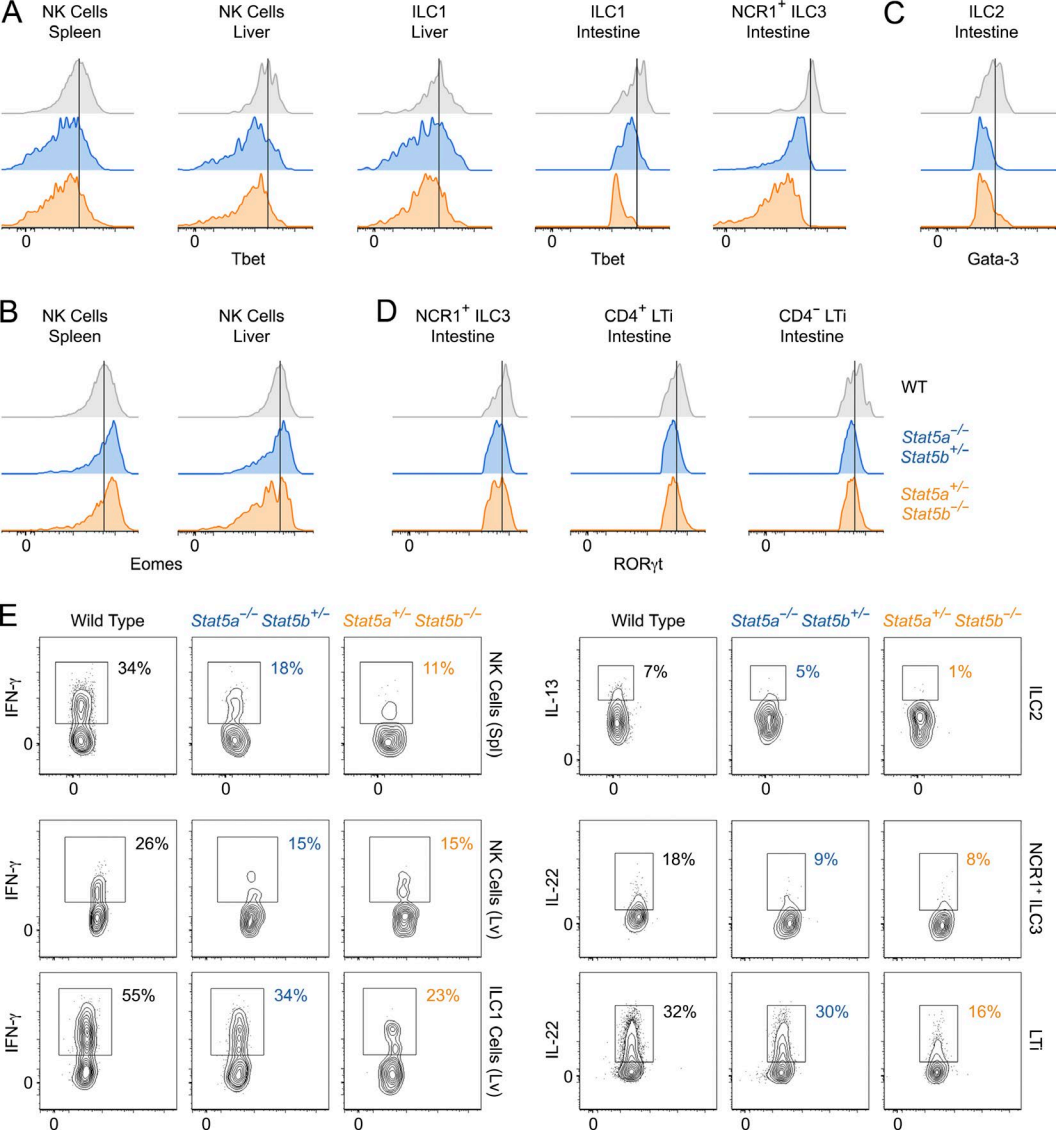
**Impact of STAT5 deficiency on ILC-defining transcription factors and cytokines.** (A–D) Flow cytometry histograms show T-BET (A), GATA-3 (B), EOMES (C), and RORγt (D) protein levels within the indicated subsets and tissues. Data are representative of four to eight experiments, depending on tissue and subset (*n* ≥ 3 mice/genotype). (E) Flow cytometry contour plots show cytokine production by ILCs from the indicated tissues. Data are representative of two to four experiments, depending on tissue and subset (*n* ≥ 3 mice/genotype).

Because all ILC subsets were diminished in one-allele STAT5A- and STAT5B-deficient mice, we next surveyed their respective LDTFs. Expression of T-BET, which is important for NK cells, ILC1, and NCR1^+^ ILC3, was notably diminished in each of these subsets and across all examined tissues (Fig. 3 A). In contrast, EOMES, which specifies only NK cells, was not reduced in the spleen and only marginally so in the liver (Fig. 3 B). GATA-3 was diminished in intestinal ILC2, and RORγt was slightly reduced in intestinal ILC3 (Fig. 3, C and D). Collectively, these data establish that STAT5 is required for optimal expression of multiple LDTFs involved in ILC specification.

Beyond lineage determination, LDTFs are important for ILC function. To assess whether reduced LDTF expression translates to functional impairment, we measured effector cytokine production in response to a STAT-independent mitogen. Whether assessing IFN-γ in NK cells and ILC1, IL-13 in ILC2, or IL-22 in ILC3, the result was clear: cytokine production was depressed in one-allele STAT5A- and STAT5B-deficient cells compared with WT controls (Fig. 3 E). Thus, beyond homeostatic defects, STAT5-deficient ILCs are functionally compromised.

### STAT5 promotes NK cell specification and function

**Figure 4.**
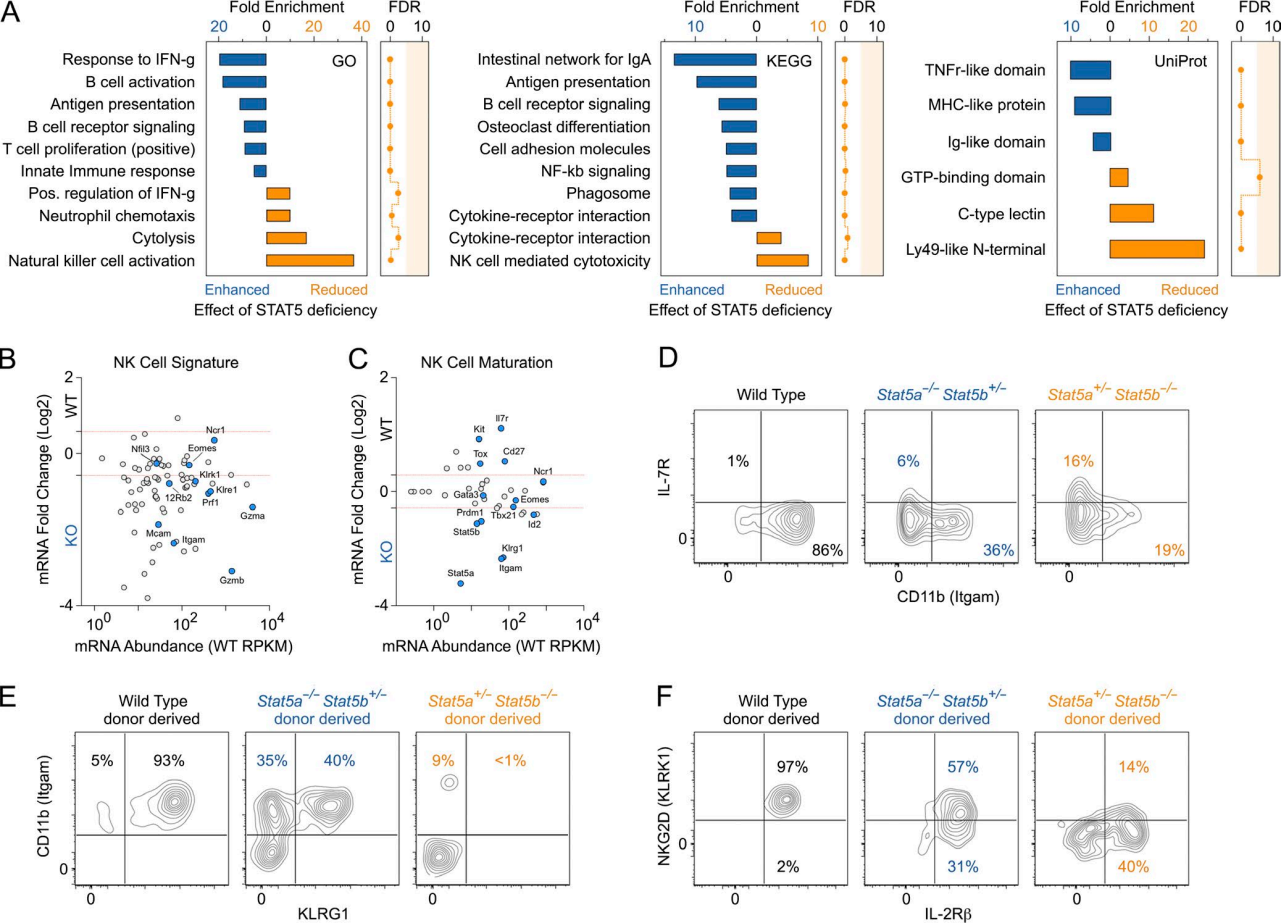
**STAT5 promotes NK cell specification and function.** (A–C) Transcriptomes were measured in splenic NK cells from WT and one-allele STAT5A-deficient mice (*Stat5a^−/−^ Stat5b^+/−^*). (A) Differentially expressed genes were segregated on the basis of whether they were enhanced or reduced in STAT5-deficient cells, and both groups were subjected to GO analysis. Bar graphs show the top enriched GO, KEGG, and UniProt terms. (B and C) MA plots show transcript abundance (x axis, mean RPKM in WT cells) and fold change (y axis, log_2_ of KO ÷ WT) for genes associated with NK cell identity (B) and NK cell maturation (C). (A–C) Two biological replicates were included per genotype. (D) Flow cytometry contour plots show percentages of immature IL7R^high^ ITGAM^low^ (top left) and mature IL7R^low^ ITGAM^high^ splenic NK cells in WT or STAT5-deficient mice. Data are representative of six experiments. (E and F) Flow cytometry contour plot show percentages of immature KLRG1^low^ ITGAM^low^ (E) or NKG2D^low^ (F) splenic NK cells derived from WT or STAT5-deficient donors in mixed bone marrow chimera experiments. Five or six donor–host pairs were assessed per genotype across two experiments.

To investigate molecular mechanisms underlying the cellular phenotypes seen in STAT5-depleted ILCs, we measured transcriptomes in NK cells, the prototypical ILC subset. For these studies, we sourced NK cells from one-allele STAT5A-deficient mice because this genotype presents obvious cellular phenotypes but retains enough residual cells for ex vivo RNA sequencing. As expected, we found that transcription of *Stat5a* was completely extinguished, transcription of *Stat5b* was decreased by half, and transcription of *Stat3*, the gene nearest to the *Stat5a/b* locus, was unaffected (Fig. S3 A). We also confirmed that, despite normal expression of all IL-15 receptor components, cytokine-driven STAT5 phosphorylation was depressed (Fig. S3 B). Overall, almost 500 genes were differentially expressed relative to WT controls, with comparable up- and down-regulated fractions (Fig. S3 C). Gene ontology (GO) analysis revealed that transcripts enhanced in one-allele STAT5A-deficient NK cells were enriched for NF-κB signaling components, particularly genes with TNF receptor–like domains, indicating that STAT5 is a negative regulator of this pathway and/or its upstream stimuli. On the other end of the spectrum, transcripts that were diminished in one-allele STAT5A-deficient NK cells were enriched for genes associated with cytotoxicity and IFN-γ production, two signature NK cell functions, as well as genes bearing C-type lectin and Ly49-like domains, two related and functionally important classes of molecules that are hallmarks of the NK cell lineage (Fig. 4 A).

To establish a role for STAT5 in specifying NK cell lineage identity, we focused on transcripts encoding NK cell signature genes, as defined by the Immunological Genome Project (Bezman et al., 2012). Remarkably, we observed a collapse of the NK cell signature in one-allele STAT5A-deficient NK cells; 52 of 76 genes were reduced compared with WT controls (Fig. 4 B). These included various members of the killer cell lectin-like receptor subfamily (e.g., *Klrk1*, *Klre1*) and cytolytic molecules (e.g., *Prf1*, *Gzmb*), consistent with the appearance of “C-type lectins” and “NK cell cytotoxicity” in our GO analysis (Fig. 4, A and B). However, although STAT5 depletion had pervasive effects, certain emblematic elements of the NK cell lineage program, including *Eomes* and *Nfil3*, were not significantly altered, indicating that there also are important STAT5-independent components.

Next, we assessed the impact of STAT5 depletion on NK cell maturation, the stepwise process by which they become functionally competent. To that end, we curated a list of maturation genes and asked whether they were dysregulated in one-allele STAT5A-deficient cells (Vosshenrich and Di Santo, 2013). This analysis revealed that genes selectively expressed by mature NK cells, such as *Gzma*, *Itgam*, *Klrk1*, and *Klrg1*, tended to be reduced, whereas those selectively expressed by immature NK cells, such as *Cd27*, *Il7r*, and *Kit*, tended to be enhanced (Fig. 4 C). We verified several of these, including Granzyme A, NKG2D (encoded by *Klrk1*), and KLRG1 (Fig. S3 D), at the protein level and confirmed that immature IL7R^high^ ITGAM^low^ NK cells are enriched in the spleens of STAT5-depleted mice (Fig. 4 D). We also confirmed that, like the overall frequency of splenic NK cells, there is a linear correlation between maturation status and total STAT5 alleles (Fig. S3 E) and that the maturation defect is cell intrinsic; one-allele STAT5A- or STAT5B-deficient donor-derived NK cells typically exhibited an immature KLRG1^low^ ITGAM^low^ or NKG2D^low^ phenotype in mixed bone marrow chimeras (Fig. 4, E and F). Thus, we conclude that STAT5 is critical for NK cell maturation and that the relative sparing of the NK cells in the bone marrow of STAT5-depleted mice reflects a pileup of immature cells that are unable to egress from their developmental niche (Figs. S1 D and S3 F).

### Defining a STAT5 signature in NK cells

**Figure 5.**
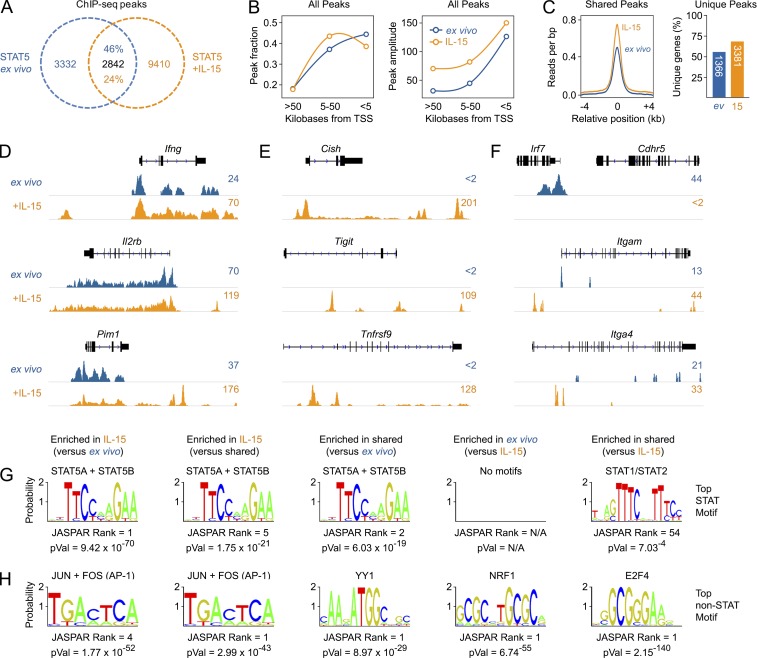
**Genome-wide distribution of STAT5 in NK cells.** (A–F) STAT5 ChIP-seq was performed in ex vivo and IL-15–treated NK cells. (A) Venn diagram denotes the total number of unique and overlapping peaks. Percentages denote the fraction of peaks shared between the two conditions. (B) Line graphs show the fraction of peaks and amplitude (mean tags per peak) for STAT5 peaks at varying distances from the nearest transcriptional start sites (TSS). (C) Histogram shows the relative amplitude (mean tags per base pair) for peaks shared between ex vivo and IL-15–treated NK cells. Bar graph shows percentage and absolute number of genes exclusively associated with peaks found in ex vivo or IL-15–treated NK cells. Denominator is all genes assigned to the 3,332 or 9,410 peaks described in A. (D–F) Genome browser tracks show STAT5 peaks at selected loci exhibiting overlapping (D), “IL-15 only” (E), or “ex vivo only” (F) distributions. (G and H) Weblogos depict the top enriched STAT (G) and non-STAT (H) transcription factor–binding motifs under STAT5 peaks from ex vivo and/or IL-15–treated NK cells. Shown are corrected p-values and rank of each motif among all database hits. (A–H) Data are presented from one of two biological replicates per condition.

STAT family TFs can influence gene expression either directly or indirectly. The former occurs through physical interactions with proximal (i.e., promoters) and/or distal (i.e., enhancers) DNA regulatory elements that enable or disable transcription of associated genes; the latter occurs through a variety of mechanisms, such as via TF intermediates that are themselves direct STAT targets (Villarino et al., 2015). Therefore, to investigate STAT5 function in ILCs, we produced genome-wide DNA binding maps in unstimulated NK cells (ex vivo), reflecting tonic signaling, and IL-15–stimulated NK cells, reflecting acute cytokine signaling. Surprisingly, we discovered widespread STAT5 occupancy in both settings, with similar proportions of gene proximal and distal binding events (Fig. 5, A and B). However, peaks found in IL-15–treated cells tended to have greater amplitude (i.e., peak height), regardless of distance to the nearest gene (Fig. 5 B), and localization was distinct; only a quarter of peaks found in IL-15–treated samples were also detected in ex vivo samples, and conversely, only half of peaks found in ex vivo samples were also detected in IL-15–treated samples (Fig. 5 A). Overlapping peaks, exemplified by the *IFNg*, *Il2rb*, and *Pim1* loci, likely reflect recent exposure to STAT5-activating cytokines by a fraction of the ex vivo population because their amplitude typically increased after exposure to IL-15 (Fig. 5, C and D). Notably, this was often accompanied by acquisition of new binding sites, suggesting that some sites can only be detected in the context of acute or saturating upstream stimuli (Fig. 5 D). Peaks found only in IL-15–treated cells illustrate the rapid recruitment of STAT5 to previously unoccupied sites and, predictably, were often found near genes whose expression is known to be regulated γc cytokines, such as *Cish*, *Tigit*, and *Tnfrsf9* (Fig. 5 E). Peaks found only in ex vivo samples had unexpected properties. About half had unique gene associations, meaning that they were detected near genes that were not bound in IL-15–treated cells, such as *Irf7*, and about half had shared gene associations, meaning that they were detected near genes that were also bound in IL-15–treated cells, such as *Itgam* and *Itga4* (Fig. 5, C and F). Both scenarios imply that STAT5 is redistributed upon exposure to IL-15 and that, overall, tonic STAT5 signaling is fundamentally distinct from acute STAT5 signaling, likely relating to differences in upstream stimuli, dosage and/or kinetics.

To gain further mechanistic insights, we compared TF-binding motifs under STAT5 peaks detected in ex vivo and/or IL-15–treated NK cells. As expected, canonical STAT-binding motifs were highly represented under “IL-15” peaks but, surprisingly, not under “ex vivo” peaks (Fig. S4 A). Head-to-head analysis yielded similar results: STAT5 was the most enriched STAT motif under “IL-15” peaks relative to “ex vivo” peaks, but the counter comparison (i.e., “ex vivo” vs. “IL-15”) yielded no STAT motif enrichment (Fig. 5 G and Table S3). Enrichment of non-STAT motifs was also distinct. AP-1 components, including JUN-FOS, BATF-JUN, and FOSL1, were the top hits under “IL-15” peaks relative to “ex vivo” peaks, whereas NRF1, E2F4, and ZBTB33 motifs were the top hits in the counter comparison (Fig. 5 H and Table S3). Both findings were corroborated by de novo motif discovery; STAT and AP-1 were the two most enriched motifs under “IL-15” peaks but were not evident under “ex vivo” peaks (not depicted). “Shared” peaks were also highly enriched for STAT5 and AP-1 (relative to “ex vivo” peaks) and exhibited unique properties when compared with “IL-15” peaks, such as YY1 motif enrichment (Fig. 5, G and H; Fig. S4 A; and Table S3). Thus, STAT5 distribution is mediated largely by canonical targeting to STAT motifs in IL-15–treated NK cells but not in ex vivo NK cells, which appears to be driven by unconventional targeting strategies.

**Figure 6.**
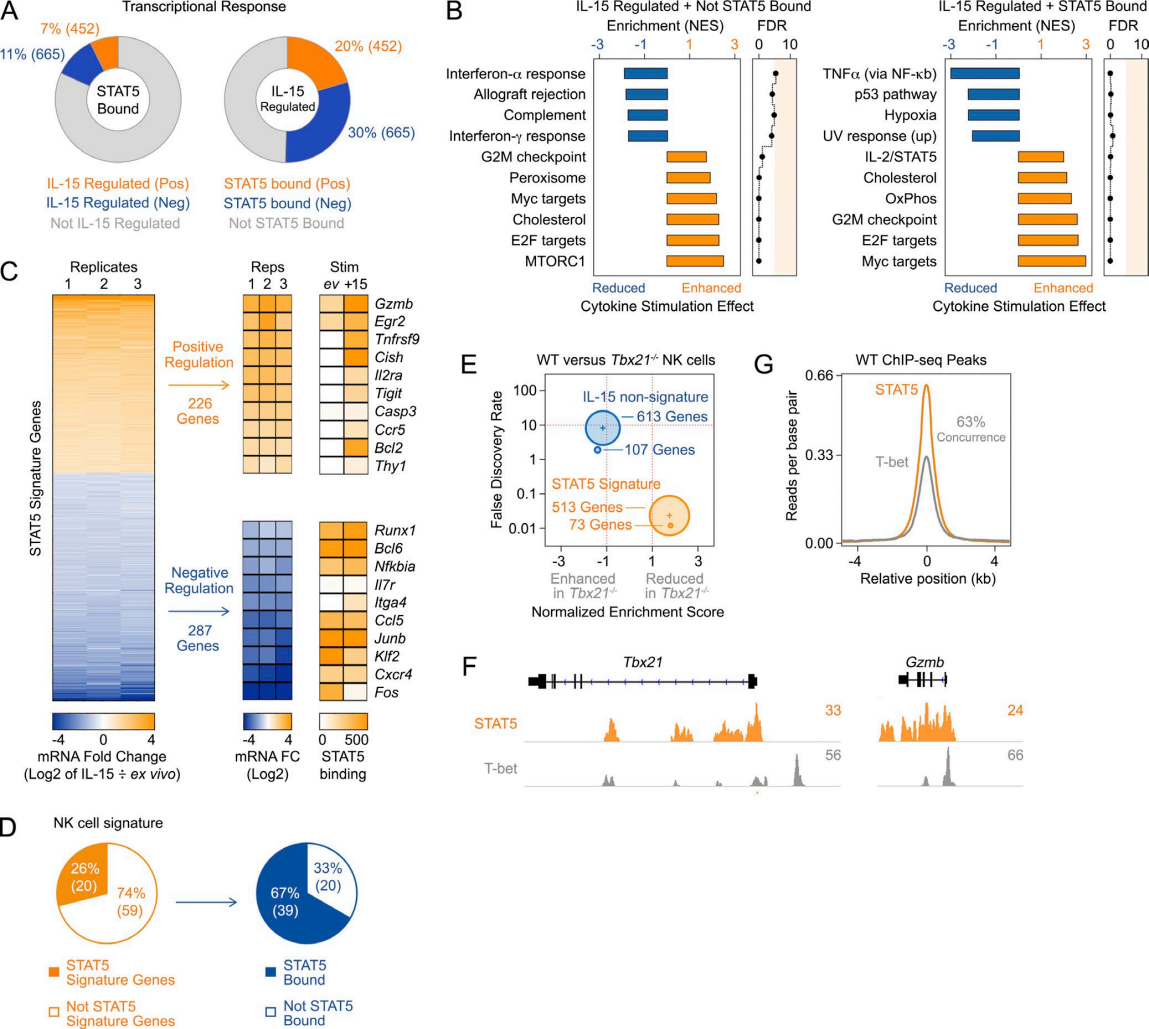
**Transcriptional activity and cooperativity of STAT5 downstream of IL-15.** Transcriptomes were compared between ex vivo and IL-15–treated NK cells. Differentially expressed genes were then segregated on the basis of whether or not STAT5-binding sites were detected near transcriptional start sites in IL-15–treated NK cells. (A) Left donut plot reports the total number of positively or negatively regulated genes from among all STAT5-bound genes. Right donut plot reports the total number of STAT5-bound, positively or negatively regulated genes from among all IL-15–regulated genes. (B) GSEA was applied to STAT5-bound and STAT5-unbound, IL-15–regulated genes. Bar graphs show hallmark pathway genes sets that were reduced or enhanced by IL-15. FDR, false discovery rate; NES, normalized enrichment score. (C) Left heat map shows the fold change in RPKM (log_2_ of IL-15–treated ÷ untreated) for all IL-15/STAT5 signature genes across three biological replicates. Right heat maps shows the RPKM fold change for a selection of signature genes and peak amplitude for the highest associated STAT5 peaks in untreated or IL-15–treated cells. (D) Left pie chart shows the number of IL-15/STAT5 signature genes among NK cell signature genes. Right pie chart shows the number of genes with nearby STAT5 peaks among NK cell signature genes that are not included in the STAT5 signature. (E) Transcriptomes were measured in IL-15–treated WT and T-BET–deficient NK cells and GSEA performed using STAT5 signature or IL-15–regulated, STAT5-unbound gene sets. Circle plot relays whether gene sets were enhanced or reduced in T-BET–deficient NK cells (y axis), along with gene set count (element size; number shown) and false discovery rate (y axis). Two biological replicates were included per genotype. (F) Genome browser tracks show STAT5 and T-BET distributions at selected loci. (G) Histogram shows the position and amplitude of T-BET-binding sites relative to STAT5-binding sites in ex vivo NK cells.

To establish a link between STAT5 binding and gene expression, we measured transcriptomic changes in IL-15–treated NK cells and cross-referenced this data set with our STAT5 distribution map, similar to prior work in T cells (Lin et al., 2012). Importantly, the majority of the >1,000 transcripts mobilized in response to IL-15 were negatively regulated, indicating that although generally regarded as a transcriptional activator, STAT5 often acts as a transcriptional repressor in NK cells (Fig. 6 A). We also noted that only ∼20% of STAT5-bound genes were transcriptionally responsive and, conversely, that only ∼50% of transcriptionally responsive genes were STAT5 bound (Fig. 6 A). Building on this latter point, we next explored the influence of STAT5-independent signaling pathways and/or TFs. To that end, we segregated IL-15–responsive genes into STAT5-bound and -unbound groups and performed gene set enrichment analysis (GSEA) using a collection of “hallmark” gene sets that includes 50 key signaling pathways and TFs. As expected, the unbound group was positively enriched for downstream targets of mTor signaling, a pathway known to mediate important IL-15–driven functions in NK cells (Marçais et al., 2014; Mao et al., 2016), whereas the STAT5-bound group was positively enriched for IL-2/STAT5 signaling (Fig. 6 B and Fig. S4 B). We also noted that NF-κB targets were negatively enriched in the STAT5-bound group, echoing our GO analysis in STAT5-deficient cells (Fig. 4 A), and that Myc target genes were positively enriched in both, suggesting that beyond its well appreciated role in promoting Myc expression (Pinz et al., 2016), STAT5 often colocalizes with this TF (Fig. 6 B and Fig. S4 B). Enrichment of Myc and IL-2/STAT5 signaling targets within the STAT5-bound group was also confirmed with an alternative pathway analysis (Fig. 6 B and Fig. S4 C).

On the basis of our integrated RNA sequencing (RNA-seq) and chromatin immunoprecipitation sequencing (ChIP-seq) analysis, we propose that at least two criteria, gene-proximal binding and transcriptional responsiveness to upstream stimuli, should be met to classify a gene as a direct STAT5 target. Following this principle, we devised two STAT5 gene signatures for NK cells, one with 513 elements and a more compact 73-element version devised using more stringent criteria for both STAT5 binding and transcriptional responsiveness (Fig. 6 C and Table S4). Importantly, one quarter of NK cell signature genes are represented, including *Gzmb*, *Klrk1*, and *Itgam*, indicating that NK cell lineage identity is, in large part, defined by the IL-15–STAT5 axis (Fig. 6 D). We also found that at least two elements, *Tnfrsf9* and *Il2ra*, are regulated by γc cytokines in other ILC subsets, suggesting the signatures may be applicable to other lineages and/or upstream stimuli (Fig. S4 D).

### Coordination between STAT5 and T-bet

During our ILC survey, we noted that the subsets most affected by STAT5 deficiency, NK cells, ILC1, and NCR1^+^ ILC3, all express T-bet. To further probe the relationship between these two TFs, we first confirmed that T-bet-deficient mice phenocopy STAT5-deficient mice in terms of accumulation and maturation of splenic NK cells (Fig. S5, A and B). Importantly, this phenocopy was evident despite normal IL-2Rβ expression and IL-15–driven STAT5 phosphorylation in T-bet-deficient NK cells (Fig. S5, C and D). Next, we measured transcriptomes in IL-15–treated WT and T-bet-deficient NK cells and applied GSEA using our 513 and 73 element STAT5 signatures. Both gene sets were highly enriched in WT NK cells, indicating that T-bet typically promotes expression of genes that are directly regulated by STAT5 (Fig. 6 E and Fig. S5 E). In contrast, complementary “unbound” gene sets were enriched in T-bet–deficient NK cells, indicating that T-bet typically inhibits expression of IL-15–regulated genes that are not direct STAT5 targets (Fig. 6 E and Fig. S5 E). We also compared genome-wide distributions and found widespread overlap between STAT5 and T-bet; 63% of STAT5 peaks had overlapping T-bet peaks, with salient examples found near genes whose expression was diminished in one-allele STAT5A-deficient NK cells, including *Tbx21* (which encodes T-bet) and *Gzmb* (Fig. 6, F and G). Collectively these data establish that STAT5 and T-bet regulate many of the same genes and suggest a collaborative relationship in NK cells.

## Discussion

The intimate relationship between γc cytokines and ILCs first became apparent >30 yr ago when it was discovered that IL-2 promotes NK cell cytotoxicity (Rosenberg and Lotze, 1986). It is now well established that γc cytokines profoundly influence NK cell biology, but the molecular basis for their varied effects remains loosely defined. A major impediment has been the fact that deletion of STAT5, the principal signaling moiety downstream of γc and its coreceptors, is incompatible with NK cell viability (Moriggl et al., 1999; Yao et al., 2006; Eckelhart et al., 2011). One approach for circumventing this issue has been to “rescue” STAT5-deficient NK cells through ectopic expression of the anti-apoptotic protein BCL2 (Minagawa et al., 2002; Gotthardt et al., 2016). These studies conclude that several key NK cell processes are subject to STAT5 but, inevitably, are confounded by STAT5-independent effects of the *Bcl2* transgene (Minagawa et al., 2002; Gotthardt et al., 2016). To overcome this caveat, we generated mice that retain one of four *Stat5* alleles, thereby restricting availability of STAT5 in NK cells, and all other ILC subsets, while avoiding the severe hematopoietic and immunological abnormalities seen in complete STAT5 KOs or BCL2-transgenic mice (Egle et al., 2004; Yao et al., 2006). By applying cytometry- and transcriptome-based phenotyping, we demonstrate that STAT5 is not only critical for NK cell homeostasis and function, as predicted by prior work in mice lacking upstream cytokines or receptors (Cao et al., 1995; Cooper et al., 2002; Colucci et al., 2003; Koka et al., 2003; Ranson et al., 2003; Vosshenrich et al., 2005; Imamura et al., 2014), but also for lineage specification and maturation. We also expanded its purview to all known ILC subsets and various types of ILTCs, in line with previous studies on upstream regulators, including Jak3, γc, IL-2Rβ, IL-7/IL-7R, and IL-15/IL-15R (Satoh-Takayama et al., 2008, 2010; Moro et al., 2010; Price et al., 2010; Vonarbourg et al., 2010; Hoyler et al., 2012; Daussy et al., 2014; Klose et al., 2014b; Merzoug et al., 2014; Vély et al., 2016; Robinette et al., 2017), and establish STAT5B as the dominant paralog in ILCs and ILTCs. Because the transcriptional output of *Stat5b* is greater than that of *Stat5a* in NK cells (Fig. S3 A), we interpret that the apparent dominance of STAT5B in this subset, and perhaps others, is due to lower STAT5 protein levels in one-allele STAT5B-deficient cells rather than widespread functional disparity between STAT5 paralogs, as shown for helper T cells (Villarino et al., 2016).

A unique benefit of our genetic model is that by retaining one allele of *Stat5a* or *Stat5b*, we were able to compare two different levels of STAT5 depletion, the former more profound than the later. This enabled us to uncover a lineage- and tissue-defined hierarchy of STAT5 dependency among ILC and ILTC subsets; with splenic and intestinal NK cells, intestinal ILC1, and intestinal NCR1^+^ ILC3 at the top of the scale, followed by intestinal ILC2, splenic NKT cells, liver NK cells, intestinal CD8αα T cells, and intestinal CD4/CD8 double-negative T cells, and ending with intestinal γδ T cells, liver ILC1, liver NKT cells, intestinal LTi cells, and bone marrow NK cells, which appear least sensitive to reduced STAT5 availability. Among ILCs, STAT5 dependency broadly correlates with each subset’s preferred γc cytokines—IL-15–sensitive NK, ILC1, and NCR1^+^ ILC3 are more affected than ILC2 and LTi (Fig. S5 F)—and with steady-state proliferation; the percentage of Ki67^+^ cells is greater for lamina propia ILC1 (7.9 ± 2.0) and NCR1^+^ ILC3 (13.8 ± 2.9) than LTi (1.9 ± 1.1). Both of these concepts, along with the vital importance of the γc-STAT5 axis, are well illustrated by recent work showing that IL-15 can foster ILC development and function in the absence of IL-7R (Robinette et al., 2017).

Depletion of ILCs in one-allele STAT5A- and STAT5B-deficient mice does not appear to be due to an early developmental block, as CLPs and CHILPs were present in both genotypes, consistent with similar findings in γc- and IL-7–deficient mice (Klose et al., 2014b). ILC2 progenitors were also present, but NK cell and ILC1 progenitors were sharply diminished, indicating that they are more sensitive to reduced STAT5 availability (Fig. S5 F). These latter findings contrast with prior work in γc- and IL-7R–deficient mice, so we interpret that (a) ILC2 progenitors can make due with limited amounts of STAT5, and (b) alternative stimuli promote STAT5-dependent NKp development in the absence of γc or IL-7R (Vosshenrich et al., 2005; Hoyler et al., 2012; Robinette et al., 2017). Notwithstanding, our data point toward postdevelopmental defects as a major disruptive force in one-allele STAT5A- and STAT5B-deficient and suggest a central role for STAT5 in promoting ILC fitness at various tissues, specifically via its ability to induce transcription of anti-apoptotic proteins such as BCL2. We also demonstrate that residual ILCs from one-allele STAT5A- and STAT5B-deficient mice are functionally compromised, indicating that, beyond homeostatic concerns, STAT5 participates in effector programing of various subsets.

Beyond affirming the importance of STAT5, our work provides valuable insights on how it operates at molecular and genomic levels. First, we discovered that genome-wide STAT5 distribution is distinct for ex vivo and IL-15–treated NK cells, which implies that its steady-state behavior, reflecting tonic signaling and/or the activity of “unphosphorylated” STAT5, is fundamentally different from acute behavior downstream of γc cytokines. Importantly, STAT-binding motifs were not enriched under peaks found in ex vivo NK cells, suggesting alternative targeting mechanisms but were highly enriched under peaks found in IL-15–treated NK cells, consistent with the long-standing idea that γc cytokines induce rapid recruitment of STAT5 to canonical binding sites. We also cross-referenced our IL-15–driven STAT5 distribution map with complementary transcriptome analyses to devise a robust STAT5-driven transcriptional signature and thus provide a strictly defined molecular rationale for its varied activities in NK cells. This strategy also allowed us to distinguish between direct STAT5 targets and alternative signaling pathways or TFs downstream of IL-15, such as mTor and Myc, thereby confirming the influence of the latter.

Like STAT5, T-bet is required for accumulation of NK cells, ILC1, and NCR1^+^ ILC3 (Townsend et al., 2004; Gordon et al., 2012; Sciumé et al., 2012; Klose et al., 2013; Rankin et al., 2013; Daussy et al., 2014). Strikingly, we found that these subsets were the most diminished in STAT5-depleted mice and that, in all cases, T-bet protein levels were lower in the residual cells. Considering that multiple studies (including the present work) have shown that STAT5 directly engages the Tbx21 locus (Liao et al., 2011; Villarino et al., 2016), we can infer that STAT5 is an important upstream regulator of T-bet in ILCs. However, we did not detect an increase in T-bet expression when WT NK cells or ILC1 were acutely exposed to γc cytokines, likely because T-bet expression was already saturated because of in vivo exposure to STAT5 stimuli and/or other instructive signals (unpublished data). Notwithstanding, our data suggest a collaborative relationship in NK cells, the prototypical ILC subset. We demonstrate that STAT5 and T-bet colocalize throughout the genome and that STAT5 signature genes are depressed in the absence of T-bet, suggesting that it may promote STAT5 activity at direct target genes. Considering that multiple T-bet^+^ innate and innate-like lymphocytes were diminished in one-allele STAT5A- and STAT5B-deficient mice, including ILC1, NCR1^+^ ILC3, NKT cells, γδ T cells, and CD8αα T cells (Townsend et al., 2004; Klose et al., 2014a; Reis et al., 2014), we propose that this relationship is important beyond NK cells.

Collectively, our data position STAT5 as a key MLTF involved in ILC specification, homeostasis, and function. Consequently, we can infer that ILC defects contribute to the panoply of symptoms seen in patients with loss-of-function STAT5B mutations, which notably exhibit a lack of circulating NK cells and barrier surface pathologies, particularly in the skin and lungs (Kanai et al., 2012). Thus, we believe that our findings have clinical relevance not only in terms of understanding STAT5 function under healthy and disease states but also as a warning for potential side effects that may accompany therapeutic intervention of this pathway.

## Materials and methods

### Experimental animals

STAT5-deficient mice were generated as described (Villarino et al., 2016). In brief, mice lacking the entire STAT5 locus (*Stat5a/b^+/−^*) were crossed with mice lacking one allele of *Stat5a* (*Stat5a^+/−^ Stat5b^+/+^*) or *Stat5b* (*Stat5a^+/+^ Stat5b^+/−^*) to produce eight allele combinations (Fig. S1 A). We refer to each genotype according to the number of *Stat5* alleles that are retained. For example, one-allele *Stat5a*-deficient mice lack both *Stat5a* alleles but retain one *Stat5b* alleles (*Stat5a^−/−^ Stat5b^+/+^*), whereas one-allele *Stat5b*-deficient mice lack both *Stat5b* alleles but retain one *Stat5a* allele (*Stat5a^+/−^ Stat5b^−/−^*). *Tbx21^−/−^* and WT C57BL/6 mice were purchased from The Jackson Laboratory. Animals were housed and handled in accordance with National Institutes of Health (NIH) guidelines and all experiments approved by the National Institute of Arthritis and Musculoskeletal and Skin Diseases (NIAMS) Animal Care and Use Committee.

### Cell purifications

Tissues were dissected from 8–16-wk-old mice and single-cell suspensions made using the following methodologies. Spleens were mechanically dissociated and red blood cells depleted by hypotonic lysis (Gibco/Life Technologies). Livers were depleted of circulating lymphocytes by flushing 5 ml PBS through hepatic portal veins, then all lobes were mechanically dissociated and lymphocytes enriched using Lymphocyte Separation Medium (Corning). Bone marrow was flushed from rear femurs and red blood cells depleted by hypotonic lysis. Lamina propia lymphocytes and intraepithelial lymphocytes were isolated from large intestines. In brief, fat deposits and Peyer’s patches were removed, luminal contents flushed and tissues sectioned into 1-cm segments. These were then bathed in HBSS containing 5 mM EDTA and 10 mM Hepes, mechanically sheared, incubated in HBSS containing 0.5 mg/ml DNase I and 0.25 mg/ml Liberase TL (Roche), and strained (100-µm filter). Lamina propia lymphocytes were enriched using 80%/40% Percoll gradient (GE Healthcare). All cell cultures were performed in RPMI-1640 medium supplemented with 10% fetal calf serum, 1% sodium pyruvate, 1% nonessential amino acids, 10 mM Hepes, 0.1% β-mercaptoethanol, 100 U/ml penicillin, and 100 mg/ml streptomycin (Life Technologies).

### Flow cytometry

Cells were stained either directly ex vivo or after in vitro culture with IL-15 (20–50 ng/ml) using fluorochrome-labeled anti-mouse antibodies recognizing the following proteins: BCL-2, CD3ε, CD4, CD8α, CD8β, CD11b (ITGAM), CD19, CD25 (IL-2Rα), CD27, CD45.1, CD45.2, CD45R (B220), CD49b (DX5), CD90.1, CD90.2, CD117 (c-Kit), CD122 (IL-2Rβ), CD127 (IL-7R), CD132 (γc), CD135 (FLT3), CD137 (TNFRSF9 or 4-1BB), CD215 (IL-15R), CD253 (TRAIL), CD278 (ICOS), Eomes, GATA-3, Granzyme A, IFN-γ, Intergin α4β7 (LPAM), IL-13, IL-22, KLRG1, NCR1 (NKp46), NK1.1, NKG2D (KLRK1), RORγt, Sca1 (Ly-6A/E), STAT5 (pTyr-694), T-BET, TCRγ, and/or TIGIT (all from eBioscience, BD Biosciences, or BioLegend). For detection of intracellular proteins, cells were fixed and permeabilized with a combination of Cytofix/Cytoperm (BD Biosciences) and 100% methanol. For cytokine production, single-cell suspensions were stimulated with Phorbol 12-myristate 13-acetate and ionomycin for 5–6 h (50 and 500 ng/ml, respectively) including Brefeldin A (10 µg/ml) for the final 3 h (all from Sigma-Aldrich). For bone marrow progenitor studies, cells were stained with biotinylated Lineage Cell Depletion kit antibody cocktail (containing anti-mouse CD5, CD45R [B220], CD11b, Ly-6G/C [Gr-1], Ly-6B.2, and Ter-119; Miltenyi Biotec) and biotinylated anti-NK1.1 and anti-NKp46 in conjunction with fluorochrome-labeled streptavidin. Phycoerythrin-labeled, αGalCer-loaded CD1d tetramers were purchased from ProImmune. Data were collected on a FACSverse cytometer (BD Biosciences) and analyzed using FlowJo (FlowJo LLC). Compiled cytometry data are presented as box plots in which horizontal lines indicate the mean of all replicates and whiskers indicate minimum and maximum values. See Fig. S2 for detailed gating strategies.

### Bone marrow chimeras

Lineage-positive cells were depleted from WT (CD45.1 or CD90.1 congenic), *Stat5a^−/−^ Stat5b^+/−^* or *Stat5a^−/−^ Stat5b^+/−^* bone marrow by negative selection using the Lineage Cell Depletion kit supplemented with biotinylated anti-NKp46 and anti-CD25 (Miltenyi Biotec). These were washed and resuspended in PBS, then WT and KO cells were mixed (WT-to-KO ratio 1:5) and intravenously transferred to sex-matched γc*^−/−^ Rag2^−/−^* hosts (500 µl total). Engraftment was measured 8–12 wk later in spleen, liver, and intestinal lamina propia.

### RNA sequencing and transcriptome analysis

Cell sorting was used to isolate CD19^−^ CD3ε^−^ NCR1^+^ CD49b^+^ NK cells from spleens of WT, *Stat5a^−/−^ Stat5b^+/^*^−^, and *Tbx21^−/−^* mice (>98% purity). Cells were processed either directly ex vivo or after in vitro culture with 20 ng/ml mouse IL-15 (eBioscience) for 12 (*Stat5a^−/−^ Stat5b^+/^*^−^ experiments) or 48 h (*Tbx21^−/−^* experiments). Equal numbers of cells (0.5–5 × 10^5^) were used per genotype/condition for each experiment. These were lysed in Trizol reagent, and total RNA was isolated by phenol-chloroform extraction with GlycoBlue as a coprecipitant (7–15 µg per sample; Life Technologies). Single-end libraries were prepared using the TruSeq RNA Sample Preparation kit v.2 and sequenced using a HiSeq 2500 instrument (Illumina). 50-bp reads (>20 million per sample) were mapped onto mouse genome build mm9 using TopHat and further processed using Cufflinks. All data sets are normalized on the basis of reads per kilobase of transcript per million mapped reads (RPKM) and purged of micro-RNAs, small nucleolar RNAs, and small Cajal body–specific RNAs. When multiple transcripts were detected for a single gene, only the most abundant (i.e., highest mean RPKM across all genotypes and conditions) was considered for downstream analyses. To minimize “fold change artifacts” caused by low-abundance transcripts, a small offset (equal to the second quartile of all data points) was added to RPKM values, and transcripts with RPKM values of <1 in all genotypes/conditions were excluded. Transcript variance was calculated using Edge-R. Transcripts were classified as differentially expressed genes (DEGs) if they exhibited >1.5-fold change and significant pairwise variance (P < 0.05) compared with ex vivo WT controls. Two or three biological replicates were sequenced per genotype or condition (Table S1). Volcano plots, bar graphs, and MA plots were generated with DataGraph (Visual Data Tools). NK cell signature and NK cell maturation gene sets used for MA plots are described in Table S4.

DAVID bioinformatics resources were used for GO analysis of DEG from *Stat5a^−/−^ Stat5b^+/^*^−^ NK cells (National Institute of Allergy and Infectious Diseases). Only lower level GO terms (GO:00) with more than threefold enrichment and a <10% false discovery rate were considered, and only the highest scoring among redundant terms were graphed. Disease-associated KEGG terms were excluded. Ingenuity Pathway Analysis was used to predict “upstream regulators” in STAT5-bound or -unbound DEGs from WT NK cells treated with IL-15 (Ingenuity Systems).

GSEA was performed as previously described (Subramanian et al., 2005). For IL-15 experiments, RPKMs for STAT5-bound or -unbound DEGs were run against GSEA hallmark gene sets (Liberzon et al., 2015). All gene sets with normalized enrichment scores greater than 2 or less than −2 in the STAT5-bound group are shown, and a matching number and pattern are presented for the STAT5-unbound data set (Fig. 6 B). Enrichment curves and member ranks are shown in Fig. S4 B. For *Tbx21^−/−^* NK cell experiments and unabridged RNA-seq data sets were used with the STAT5 signature gene sets detailed in Table S4. A circle plot was generated with DataGraph (Fig. 6 G). All RNA-seq data are available from the NCBI Gene Expression Omnibus under accession no. GSE100674.

### Chromatin immunoprecipitation and DNA sequencing

10^7^ NK cells were purified from spleens of WT mice by magnetic bead separation (90% purity; negative selection kit by Miltenyi Biotec) and fixed with 1% formaldehyde either directly ex vivo or after treatment with mouse IL-15 (2 h, 20 ng/ml). Cells were then lysed and sonicated before immunoprecipitation with rabbit anti-mouse pan-STAT5 antibody (ab7969; Abcam). Recovered STAT5-bound DNA fragments, along with unprecipitated “input controls,” were blunt-end ligated to adaptors and single-end libraries prepared using the NEBNext ChIP-Seq Library Prep for Illumina kit (New England Biolabs). Sequencing was performed on a HiSeq 2500 instrument (50 cycles; Illumina), and Bowtie was used to map >10 million nonredundant 50-bp reads to mouse genome build mm9. Peaks with more than twofold enrichment over background, p-values < 0.00001, and false discovery rate < 5% were called with MACS 1.4.2 using input controls as background reference. Two biological replicates were sequenced per condition (Table S2). bamCorrelate from deepTools v.1.5 was used to calculate Spearman’s rank correlation coefficients as a measure of interreplicate variability (ex vivo r = 0.8737, IL-15 r = 0.8737, P < 2.2 × 10^−16^ for both). Downstream analyses were performed separately; data shown are from the second replicate. ChIP-seq data for T-BET in ex vivo NK cells have been published (Shih et al., 2016).

The IL-15/STAT5 signature (513 elements) includes genes that (a) are STAT5-bound based on ChIP-seq from IL-15–treated NK cells (peak detected within gene body or ± 50 kb from transcriptional start site) and (b) are transcriptionally regulated by IL-15 on the basis of RNA-seq comparison of ex vivo and IL-15–treated NK cells (more than twofold change in RPKM; P < 0.05). The compact IL-15/STAT5 signature (73 elements) includes genes that (a) exhibit high amplitude STAT5 peaks (tag count for at least one gene-associated peak is within the top 25% of all peaks) and (b) exhibit more than threefold change in RPKM downstream of IL-15 (P < 0.05). Only genes with mean RPKM > 2 in either ex vivo or IL-15–treated NK cells are included (Table S4).

Direct comparison of peak distribution across experimental groups was done with PAPST and annotated to the nearest gene using HOMER. Peaks are assigned to genes if they occur within introns, exons, and/or <50 kb from transcriptional start sites. Peak distribution was further analyzed, and histograms were generated using ChAsE. Known and de novo TF motif enrichment analysis was performed using the AME and DREME tools of the MEME suite, focusing on JASPAR database outputs. Line graphs, donut plots, and pie charts were generated using DataGraph. Heat maps were generated using Multi Experiment Viewer (J. Craig Venter Institute). Genome browser tracks are displayed with the Integrative Genomics Viewer (Broad Institute). All ChIP-seq data are available from the NCBI Gene Expression Omnibus under accession no. GSE100674.

### Statistics

Unpaired homoscedastic Student’s *t* test was used to quantify statistical deviation between experiential groups. Asterisks denote significant differences (P < 0.05) compared with WT controls.

### Online supplemental material

Fig. S1 details the mouse models used in this study and shows the frequency of splenic NK cells in all genotypes. Fig. S2 shows flow cytometry gating strategies for NK cells, ILC subsets, ILTCs, and ILC precursors. Fig. S3 shows transcriptome and flow cytometry data comparing WT and one-allele STAT5A-deficient NK cells. Fig. S4 shows enrichment of STAT-binding motifs under STAT5 ChIP-seq peaks, GSEA enrichment plots for hallmark genes, upstream regulator analysis for STAT5-bound and -unbound DEGs, and flow cytometry data for STAT5 signature genes in various ILC subsets. Fig. S5 shows flow cytometry–based phenotyping of *Tbx21^−/−^* NK cells, GSEA enrichment plots for STAT5 signature genes in WT and *Tbx21^−/−^* NK cells, and a cartoon illustrating the hierarchy of STAT5 dependency among ILC subsets and precursors. Table S1 is a spreadsheet containing RPKM measurements, fold change calculations, and p-values for RNA-seq experiments. Table S2 is a spreadsheet containing annotated STAT5 ChIP-seq data. Table S3 is a spreadsheet containing enrichment ranks and p-values for TF-binding motifs found under STAT5 peaks. Table S4 is a spreadsheet defining the STAT5 gene signature in NK cells and all gene sets used for transcriptome analysis. Tables S1–S4 are provided as Excel files.

## Supplementary Material

Supplemental Materials (PDF)

Tables S1-S4 (zipped Excel files)

## References

[bib1] Artis D., and Spits H. 2015 The biology of innate lymphoid cells. Nature. 517:293–301. 10.1038/nature14189 25592534

[bib2] Bezman N.A., Kim C.C., Sun J.C., Min-Oo G., Hendricks D.W., Kamimura Y., Best J.A., Goldrath A.W., Lanier L.L., Gautier E.L., Immunological Genome Project Consortium 2012 Molecular definition of the identity and activation of natural killer cells. Nat. Immunol. 13:1000–1009. 10.1038/ni.2395 22902830 PMC3572860

[bib3] Boesteanu A., Silva A.D., Nakajima H., Leonard W.J., Peschon J.J., and Joyce S. 1997 Distinct roles for signals relayed through the common cytokine receptor γ chain and interleukin 7 receptor α chain in natural T cell development. J. Exp. Med. 186:331–336. 10.1084/jem.186.2.331 9221763 PMC2198975

[bib4] Cao X., Shores E.W., Hu-Li J., Anver M.R., Kelsall B.L., Russell S.M., Drago J., Noguchi M., Grinberg A., Bloom E.T., 1995 Defective lymphoid development in mice lacking expression of the common cytokine receptor gamma chain. Immunity. 2:223–238. 10.1016/1074-7613(95)90047-0 7697543

[bib5] Cheroutre H., Lambolez F., and Mucida D. 2011 The light and dark sides of intestinal intraepithelial lymphocytes. Nat. Rev. Immunol. 11:445–456. 10.1038/nri3007 21681197 PMC3140792

[bib6] Colucci F., Caligiuri M.A., and Di Santo J.P. 2003 What does it take to make a natural killer? Nat. Rev. Immunol. 3:413–425. 10.1038/nri1088 12766763

[bib7] Constantinides M.G., McDonald B.D., Verhoef P.A., and Bendelac A. 2014 A committed precursor to innate lymphoid cells. Nature. 508:397–401. 10.1038/nature13047 24509713 PMC4003507

[bib8] Constantinides M.G., Gudjonson H., McDonald B.D., Ishizuka I.E., Verhoef P.A., Dinner A.R., and Bendelac A. 2015 PLZF expression maps the early stages of ILC1 lineage development. Proc. Natl. Acad. Sci. USA. 112:5123–5128. 10.1073/pnas.1423244112 25838284 PMC4413309

[bib9] Cooper M.A., Bush J.E., Fehniger T.A., VanDeusen J.B., Waite R.E., Liu Y., Aguila H.L., and Caligiuri M.A. 2002 In vivo evidence for a dependence on interleukin 15 for survival of natural killer cells. Blood. 100:3633–3638. 10.1182/blood-2001-12-0293 12393617

[bib10] Daussy C., Faure F., Mayol K., Viel S., Gasteiger G., Charrier E., Bienvenu J., Henry T., Debien E., Hasan U.A., 2014 T-bet and Eomes instruct the development of two distinct natural killer cell lineages in the liver and in the bone marrow. J. Exp. Med. 211:563–577. 10.1084/jem.20131560 24516120 PMC3949572

[bib11] De Obaldia M.E., and Bhandoola A. 2015 Transcriptional regulation of innate and adaptive lymphocyte lineages. Annu. Rev. Immunol. 33:607–642. 10.1146/annurev-immunol-032414-112032 25665079

[bib12] Diefenbach A., Colonna M., and Koyasu S. 2014 Development, differentiation, and diversity of innate lymphoid cells. Immunity. 41:354–365. 10.1016/j.immuni.2014.09.005 25238093 PMC4171710

[bib13] DiSanto J.P., Müller W., Guy-Grand D., Fischer A., and Rajewsky K. 1995 Lymphoid development in mice with a targeted deletion of the interleukin 2 receptor gamma chain. Proc. Natl. Acad. Sci. USA. 92:377–381. 10.1073/pnas.92.2.377 7831294 PMC42743

[bib14] Eckelhart E., Warsch W., Zebedin E., Simma O., Stoiber D., Kolbe T., Rülicke T., Mueller M., Casanova E., and Sexl V. 2011 A novel Ncr1-Cre mouse reveals the essential role of STAT5 for NK-cell survival and development. Blood. 117:1565–1573. 10.1182/blood-2010-06-291633 21127177

[bib15] Egle A., Harris A.W., Bath M.L., O’Reilly L., and Cory S. 2004 VavP-Bcl2 transgenic mice develop follicular lymphoma preceded by germinal center hyperplasia. Blood. 103:2276–2283. 10.1182/blood-2003-07-2469 14630790

[bib16] Ettersperger J., Montcuquet N., Malamut G., Guegan N., Lopez-Lastra S., Gayraud S., Reimann C., Vidal E., Cagnard N., Villarese P., 2016 Interleukin-15-dependent T-cell-like innate intraepithelial lymphocytes develop in the intestine and transform into lymphomas in celiac disease. Immunity. 45:610–625. 10.1016/j.immuni.2016.07.018 27612641

[bib17] Gordon S.M., Chaix J., Rupp L.J., Wu J., Madera S., Sun J.C., Lindsten T., and Reiner S.L. 2012 The transcription factors T-bet and Eomes control key checkpoints of natural killer cell maturation. Immunity. 36:55–67. 10.1016/j.immuni.2011.11.016 22261438 PMC3381976

[bib18] Gotthardt D., Putz E.M., Grundschober E., Prchal-Murphy M., Straka E., Kudweis P., Heller G., Bago-Horvath Z., Witalisz-Siepracka A., Cumaraswamy A.A., 2016 STAT5 is a key regulator in NK cells and acts as a molecular switch from tumor surveillance to tumor promotion. Cancer Discov. 6:414–429. 10.1158/2159-8290.CD-15-0732 26873347

[bib19] He Y.W., and Malek T.R. 1996 Interleukin-7 receptor α is essential for the development of γ Δ + T cells, but not natural killer cells. J. Exp. Med. 184:289–293. 10.1084/jem.184.1.289 8691145 PMC2192680

[bib20] Hoelbl A., Kovacic B., Kerenyi M.A., Simma O., Warsch W., Cui Y., Beug H., Hennighausen L., Moriggl R., and Sexl V. 2006 Clarifying the role of Stat5 in lymphoid development and Abelson-induced transformation. Blood. 107:4898–4906. 10.1182/blood-2005-09-3596 16493008 PMC2875852

[bib21] Hoyler T., Klose C.S.N., Souabni A., Turqueti-Neves A., Pfeifer D., Rawlins E.L., Voehringer D., Busslinger M., and Diefenbach A. 2012 The transcription factor GATA-3 controls cell fate and maintenance of type 2 innate lymphoid cells. Immunity. 37:634–648. 10.1016/j.immuni.2012.06.020 23063333 PMC3662874

[bib22] Huntington N.D., Puthalakath H., Gunn P., Naik E., Michalak E.M., Smyth M.J., Tabarias H., Degli-Esposti M.A., Dewson G., Willis S.N., 2007 Interleukin 15-mediated survival of natural killer cells is determined by interactions among Bim, Noxa and Mcl-1. Nat. Immunol. 8:856–863. 10.1038/ni1487 17618288 PMC2951739

[bib23] Imada K., Bloom E.T., Nakajima H., Horvath-Arcidiacono J.A., Udy G.B., Davey H.W., and Leonard W.J. 1998 Stat5b is essential for natural killer cell-mediated proliferation and cytolytic activity. J. Exp. Med. 188:2067–2074. 10.1084/jem.188.11.2067 9841920 PMC2212377

[bib24] Imamura M., Shook D., Kamiya T., Shimasaki N., Chai S.M.H., Coustan-Smith E., Imai C., and Campana D. 2014 Autonomous growth and increased cytotoxicity of natural killer cells expressing membrane-bound interleukin-15. Blood. 124:1081–1088. 10.1182/blood-2014-02-556837 25006133

[bib25] Kanai T., Jenks J., and Nadeau K.C. 2012 The STAT5b pathway defect and autoimmunity. Front. Immunol. 3:234 10.3389/fimmu.2012.00234 22912632 PMC3418548

[bib26] Kennedy M.K., Glaccum M., Brown S.N., Butz E.A., Viney J.L., Embers M., Matsuki N., Charrier K., Sedger L., Willis C.R., 2000 Reversible defects in natural killer and memory CD8 T cell lineages in interleukin 15-deficient mice. J. Exp. Med. 191:771–780. 10.1084/jem.191.5.771 10704459 PMC2195858

[bib27] Klose C.S.N., Kiss E.A., Schwierzeck V., Ebert K., Hoyler T., d’Hargues Y., Göppert N., Croxford A.L., Waisman A., Tanriver Y., and Diefenbach A. 2013 A T-bet gradient controls the fate and function of CCR6-RORγt+ innate lymphoid cells. Nature. 494:261–265. 10.1038/nature11813 23334414

[bib28] Klose C.S.N., Blatz K., d’Hargues Y., Hernandez P.P., Kofoed-Nielsen M., Ripka J.F., Ebert K., Arnold S.J., Diefenbach A., Palmer E., and Tanriver Y. 2014 a The transcription factor T-bet is induced by IL-15 and thymic agonist selection and controls CD8αα(+) intraepithelial lymphocyte development. Immunity. 41:230–243. 10.1016/j.immuni.2014.06.018 25148024

[bib29] Klose C.S.N., Flach M., Möhle L., Rogell L., Hoyler T., Ebert K., Fabiunke C., Pfeifer D., Sexl V., Fonseca-Pereira D., 2014 b Differentiation of type 1 ILCs from a common progenitor to all helper-like innate lymphoid cell lineages. Cell. 157:340–356. 10.1016/j.cell.2014.03.030 24725403

[bib30] Koka R., Burkett P.R., Chien M., Chai S., Chan F., Lodolce J.P., Boone D.L., and Ma A. 2003 Interleukin (IL)-15R[α]-deficient natural killer cells survive in normal but not IL-15R[α]-deficient mice. J. Exp. Med. 197:977–984. 10.1084/jem.20021836 12695489 PMC2193874

[bib31] Liao W., Lin J.-X., Wang L., Li P., and Leonard W.J. 2011 Modulation of cytokine receptors by IL-2 broadly regulates differentiation into helper T cell lineages. Nat. Immunol. 12:551–559. 10.1038/ni.2030 21516110 PMC3304099

[bib32] Liberzon A., Birger C., Thorvaldsdóttir H., Ghandi M., Mesirov J.P., and Tamayo P. 2015 The Molecular Signatures Database (MSigDB) hallmark gene set collection. Cell Syst. 1:417–425. 10.1016/j.cels.2015.12.004 26771021 PMC4707969

[bib33] Lin J.-X., Li P., Liu D., Jin H.-T., He J., Ata Ur Rasheed M., Rochman Y., Wang L., Cui K., Liu C., 2012 Critical Role of STAT5 transcription factor tetramerization for cytokine responses and normal immune function. Immunity. 36:586–599. 10.1016/j.immuni.2012.02.017 22520852 PMC3551341

[bib34] Lodolce J.P., Boone D.L., Chai S., Swain R.E., Dassopoulos T., Trettin S., and Ma A. 1998 IL-15 receptor maintains lymphoid homeostasis by supporting lymphocyte homing and proliferation. Immunity. 9:669–676. 10.1016/S1074-7613(00)80664-0 9846488

[bib35] Maki K., Sunaga S., Komagata Y., Kodaira Y., Mabuchi A., Karasuyama H., Yokomuro K., Miyazaki J.I., and Ikuta K. 1996 Interleukin 7 receptor-deficient mice lack gammadelta T cells. Proc. Natl. Acad. Sci. USA. 93:7172–7177. 10.1073/pnas.93.14.7172 8692964 PMC38955

[bib36] Mao Y., van Hoef V., Zhang X., Wennerberg E., Lorent J., Witt K., Masvidal L., Liang S., Murray S., Larsson O., 2016 IL-15 activates mTOR and primes stress-activated gene expression leading to prolonged antitumor capacity of NK cells. Blood. 128:1475–1489. 10.1182/blood-2016-02-698027 27465917 PMC5025899

[bib37] Marçais A., Cherfils-Vicini J., Viant C., Degouve S., Viel S., Fenis A., Rabilloud J., Mayol K., Tavares A., Bienvenu J., 2014 The metabolic checkpoint kinase mTOR is essential for IL-15 signaling during the development and activation of NK cells. Nat. Immunol. 15:749–757. 10.1038/ni.2936 24973821 PMC4110708

[bib38] McKenzie A.N.J., Spits H., and Eberl G. 2014 Innate lymphoid cells in inflammation and immunity. Immunity. 41:366–374. 10.1016/j.immuni.2014.09.006 25238094

[bib39] Merzoug L.B., Marie S., Satoh-Takayama N., Lesjean S., Albanesi M., Luche H., Fehling H.J., Di Santo J.P., and Vosshenrich C.A.J. 2014 Conditional ablation of NKp46+ cells using a novel Ncr1(greenCre) mouse strain: NK cells are essential for protection against pulmonary B16 metastases. Eur. J. Immunol. 44:3380–3391. 10.1002/eji.201444643 25142413

[bib40] Minagawa M., Watanabe H., Miyaji C., Tomiyama K., Shimura H., Ito A., Ito M., Domen J., Weissman I.L., and Kawai K. 2002 Enforced expression of Bcl-2 restores the number of NK cells, but does not rescue the impaired development of NKT cells or intraepithelial lymphocytes, in IL-2/IL-15 receptor beta-chain-deficient mice. J. Immunol. 169:4153–4160. 10.4049/jimmunol.169.8.4153 12370344

[bib41] Moriggl R., Topham D.J., Teglund S., Sexl V., McKay C., Wang D., Hoffmeyer A., van Deursen J., Sangster M.Y., Bunting K.D., 1999 Stat5 is required for IL-2-induced cell cycle progression of peripheral T cells. Immunity. 10:249–259. 10.1016/S1074-7613(00)80025-4 10072077

[bib42] Moro K., Yamada T., Tanabe M., Takeuchi T., Ikawa T., Kawamoto H., Furusawa J., Ohtani M., Fujii H., and Koyasu S. 2010 Innate production of T(H)2 cytokines by adipose tissue-associated c-Kit(+)Sca-1(+) lymphoid cells. Nature. 463:540–544. 10.1038/nature08636 20023630

[bib43] Park S.Y., Saijo K., Takahashi T., Osawa M., Arase H., Hirayama N., Miyake K., Nakauchi H., Shirasawa T., and Saito T. 1995 Developmental defects of lymphoid cells in Jak3 kinase-deficient mice. Immunity. 3:771–782. 10.1016/1074-7613(95)90066-7 8777722

[bib44] Pinz S., Unser S., and Rascle A. 2016 Signal transducer and activator of transcription STAT5 is recruited to c-Myc super-enhancer. BMC Mol. Biol. 17:10 10.1186/s12867-016-0063-y 27074708 PMC4831086

[bib45] Price A.E., Liang H.-E., Sullivan B.M., Reinhardt R.L., Eisley C.J., Erle D.J., and Locksley R.M. 2010 Systemically dispersed innate IL-13-expressing cells in type 2 immunity. Proc. Natl. Acad. Sci. USA. 107:11489–11494. 10.1073/pnas.1003988107 20534524 PMC2895098

[bib46] Rankin L.C., Groom J.R., Chopin M., Herold M.J., Walker J.A., Mielke L.A., McKenzie A.N.J., Carotta S., Nutt S.L., and Belz G.T. 2013 The transcription factor T-bet is essential for the development of NKp46+ innate lymphocytes via the Notch pathway. Nat. Immunol. 14:389–395. 10.1038/ni.2545 23455676 PMC4076532

[bib47] Ranson T., Vosshenrich C.A.J., Corcuff E., Richard O., Laloux V., Lehuen A., and Di Santo J.P. 2003 IL-15 availability conditions homeostasis of peripheral natural killer T cells. Proc. Natl. Acad. Sci. USA. 100:2663–2668. 10.1073/pnas.0535482100 12598649 PMC151397

[bib48] Reis B.S., Hoytema van Konijnenburg D.P., Grivennikov S.I., and Mucida D. 2014 Transcription factor T-bet regulates intraepithelial lymphocyte functional maturation. Immunity. 41:244–256. 10.1016/j.immuni.2014.06.017 25148025 PMC4287410

[bib49] Robinette M.L., Bando J.K., Song W., Ulland T.K., Gilfillan S., and Colonna M. 2017 IL-15 sustains IL-7R-independent ILC2 and ILC3 development. Nat. Commun. 8:14601 10.1038/ncomms14601 28361874 PMC5380969

[bib50] Rosenberg S.A., and Lotze M.T. 1986 Cancer immunotherapy using interleukin-2 and interleukin-2-activated lymphocytes. Annu. Rev. Immunol. 4:681–709. 10.1146/annurev.iy.04.040186.003341 3518753

[bib51] Sathe P., Delconte R.B., Souza-Fonseca-Guimaraes F., Seillet C., Chopin M., Vandenberg C.J., Rankin L.C., Mielke L.A., Vikstrom I., Kolesnik T.B., 2014 Innate immunodeficiency following genetic ablation of Mcl1 in natural killer cells. Nat. Commun. 5:4539 10.1038/ncomms5539 25119382

[bib52] Satoh-Takayama N., Vosshenrich C.A.J., Lesjean-Pottier S., Sawa S., Lochner M., Rattis F., Mention J.-J., Thiam K., Cerf-Bensussan N., Mandelboim O., 2008 Microbial flora drives interleukin 22 production in intestinal NKp46+ cells that provide innate mucosal immune defense. Immunity. 29:958–970. 10.1016/j.immuni.2008.11.001 19084435

[bib53] Satoh-Takayama N., Lesjean-Pottier S., Vieira P., Sawa S., Eberl G., Vosshenrich C.A.J., and Di Santo J.P. 2010 IL-7 and IL-15 independently program the differentiation of intestinal CD3-NKp46+ cell subsets from Id2-dependent precursors. J. Exp. Med. 207:273–280. 10.1084/jem.20092029 20142427 PMC2822619

[bib54] Sciumé G., Hirahara K., Takahashi H., Laurence A., Villarino A.V., Singleton K.L., Spencer S.P., Wilhelm C., Poholek A.C., Vahedi G., 2012 Distinct requirements for T-bet in gut innate lymphoid cells. J. Exp. Med. 209:2331–2338. 10.1084/jem.20122097 23209316 PMC3526352

[bib55] Seillet C., Rankin L.C., Groom J.R., Mielke L.A., Tellier J., Chopin M., Huntington N.D., Belz G.T., and Carotta S. 2014 Nfil3 is required for the development of all innate lymphoid cell subsets. J. Exp. Med. 211:1733–1740. 10.1084/jem.20140145 25092873 PMC4144736

[bib56] Serafini N., Klein Wolterink R.G.J., Satoh-Takayama N., Xu W., Vosshenrich C.A.J., Hendriks R.W., and Di Santo J.P. 2014 Gata3 drives development of RORγt+ group 3 innate lymphoid cells. J. Exp. Med. 211:199–208. 10.1084/jem.20131038 24419270 PMC3920560

[bib57] Serafini N., Vosshenrich C.A.J., and Di Santo J.P. 2015 Transcriptional regulation of innate lymphoid cell fate. Nat. Rev. Immunol. 15:415–428. 10.1038/nri3855 26065585

[bib58] Shenoy A.R., Kirschnek S., and Häcker G. 2014 IL-15 regulates Bcl-2 family members Bim and Mcl-1 through JAK/STAT and PI3K/AKT pathways in T cells. Eur. J. Immunol. 44:2500–2507. 10.1002/eji.201344238 24825007

[bib59] Shih H.-Y., Sciumè G., Poholek A.C., Vahedi G., Hirahara K., Villarino A.V., Bonelli M., Bosselut R., Kanno Y., Muljo S.A., and O’Shea J.J. 2014 Transcriptional and epigenetic networks of helper T and innate lymphoid cells. Immunol. Rev. 261:23–49. 10.1111/imr.12208 25123275 PMC4321863

[bib60] Shih H.-Y., Sciumè G., Mikami Y., Guo L., Sun H.-W., Brooks S.R., Urban J.F. Jr., Davis F.P., Kanno Y., and O’Shea J.J. 2016 Developmental acquisition of regulomes underlies innate lymphoid cell functionality. Cell. 165:1120–1133. 10.1016/j.cell.2016.04.029 27156451 PMC4874839

[bib61] Spits H., Bernink J.H., and Lanier L. 2016 NK cells and type 1 innate lymphoid cells: partners in host defense. Nat. Immunol. 17:758–764. 10.1038/ni.3482 27328005

[bib62] Subramanian A., Tamayo P., Mootha V.K., Mukherjee S., Ebert B.L., Gillette M.A., Paulovich A., Pomeroy S.L., Golub T.R., Lander E.S., and Mesirov J.P. 2005 Gene set enrichment analysis: a knowledge-based approach for interpreting genome-wide expression profiles. Proc. Natl. Acad. Sci. USA. 102:15545–15550. 10.1073/pnas.0506580102 16199517 PMC1239896

[bib63] Suzuki H., Duncan G.S., Takimoto H., and Mak T.W. 1997 Abnormal development of intestinal intraepithelial lymphocytes and peripheral natural killer cells in mice lacking the IL-2 receptor β chain. J. Exp. Med. 185:499–506. 10.1084/jem.185.3.499 9053450 PMC2196040

[bib64] Townsend M.J., Weinmann A.S., Matsuda J.L., Salomon R., Farnham P.J., Biron C.A., Gapin L., and Glimcher L.H. 2004 T-bet regulates the terminal maturation and homeostasis of NK and Valpha14i NKT cells. Immunity. 20:477–494. 10.1016/S1074-7613(04)00076-7 15084276

[bib65] Vély F., Barlogis V., Vallentin B., Neven B., Piperoglou C., Ebbo M., Perchet T., Petit M., Yessaad N., Touzot F., 2016 Evidence of innate lymphoid cell redundancy in humans. Nat. Immunol. 17:1291–1299. 10.1038/ni.3553 27618553 PMC5074366

[bib66] Viant C., Guia S., Hennessy R.J., Rautela J., Pham K., Bernat C., Goh W., Jiao Y., Delconte R., Roger M., 2017 Cell cycle progression dictates the requirement for BCL2 in natural killer cell survival. J. Exp. Med. 214:491–510. 10.1084/jem.20160869 28057804 PMC5294858

[bib67] Villarino A.V., Kanno Y., Ferdinand J.R., and O’Shea J.J. 2015 Mechanisms of Jak/STAT signaling in immunity and disease. J. Immunol. 194:21–27. 10.4049/jimmunol.1401867 25527793 PMC4524500

[bib68] Villarino A., Laurence A., Robinson G.W., Bonelli M., Dema B., Afzali B., Shih H.-Y., Sun H.-W., Brooks S.R., Hennighausen L., 2016 Signal transducer and activator of transcription 5 (STAT5) paralog dose governs T cell effector and regulatory functions. eLife. 5:e08384 10.7554/eLife.08384 26999798 PMC4856466

[bib69] Vonarbourg C., and Diefenbach A. 2012 Multifaceted roles of interleukin-7 signaling for the development and function of innate lymphoid cells. Semin. Immunol. 24:165–174. 10.1016/j.smim.2012.03.002 22541512

[bib70] Vonarbourg C., Mortha A., Bui V.L., Hernandez P.P., Kiss E.A., Hoyler T., Flach M., Bengsch B., Thimme R., Hölscher C., 2010 Regulated expression of nuclear receptor RORγt confers distinct functional fates to NK cell receptor-expressing RORγt(+) innate lymphocytes. Immunity. 33:736–751. 10.1016/j.immuni.2010.10.017 21093318 PMC3042726

[bib71] Vosshenrich C.A., and Di Santo J.P. 2013 Developmental programming of natural killer and innate lymphoid cells. Curr. Opin. Immunol. 25:130–138. 10.1016/j.coi.2013.02.002 23490162

[bib72] Vosshenrich C.A., Ranson T., Samson S.I., Corcuff E., Colucci F., Rosmaraki E.E., and Di Santo J.P. 2005 Roles for common cytokine receptor γ-chain-dependent cytokines in the generation, differentiation, and maturation of NK cell precursors and peripheral NK cells in vivo. J. Immunol. 174:1213–1221. 10.4049/jimmunol.174.3.1213 15661875

[bib73] Wang F., Tian Z., and Wei H. 2015 Genomic expression profiling of NK cells in health and disease. Eur. J. Immunol. 45:661–678. 10.1002/eji.201444998 25476835

[bib74] Xu W., Domingues R.G., Fonseca-Pereira D., Ferreira M., Ribeiro H., Lopez-Lastra S., Motomura Y., Moreira-Santos L., Bihl F., Braud V., 2015 NFIL3 orchestrates the emergence of common helper innate lymphoid cell precursors. Cell Reports. 10:2043–2054. 10.1016/j.celrep.2015.02.057 25801035

[bib75] Yagi R., Zhong C., Northrup D.L., Yu F., Bouladoux N., Spencer S., Hu G., Barron L., Sharma S., Nakayama T., 2014 The transcription factor GATA3 is critical for the development of all IL-7Rα-expressing innate lymphoid cells. Immunity. 40:378–388. 10.1016/j.immuni.2014.01.012 24631153 PMC4026797

[bib76] Yao Z., Cui Y., Watford W.T., Bream J.H., Yamaoka K., Hissong B.D., Li D., Durum S.K., Jiang Q., Bhandoola A., 2006 Stat5a/b are essential for normal lymphoid development and differentiation. Proc. Natl. Acad. Sci. USA. 103:1000–1005. 10.1073/pnas.0507350103 16418296 PMC1327727

[bib77] Yu X., Wang Y., Deng M., Li Y., Ruhn K.A., Zhang C.C., and Hooper L.V. 2014 The basic leucine zipper transcription factor NFIL3 directs the development of a common innate lymphoid cell precursor. eLife. 3 10.7554/eLife.04406 PMC4356142 25310240

[bib78] Zook E.C., and Kee B.L. 2016 Development of innate lymphoid cells. Nat. Immunol. 17:775–782. 10.1038/ni.3481 27328007

