# Supplementary material for: Subset- and tissue-defined STAT5 thresholds control homeostasis and function of innate lymphoid cells
Source: J Exp Med. 2017 Oct 2;214(10):2999–3014. doi: 10.1084/jem.20150907 (PMC5626390; doi:10.1084/jem.20150907)
Supplement: Supplemental Materials (PDF) [file JEM_20150907_sm.pdf]

The Journal of Experimental Medicine

SUPPLEMENTAL MATERIAL

Villarino et al., https://doi.org/10.1084/jem.20150907

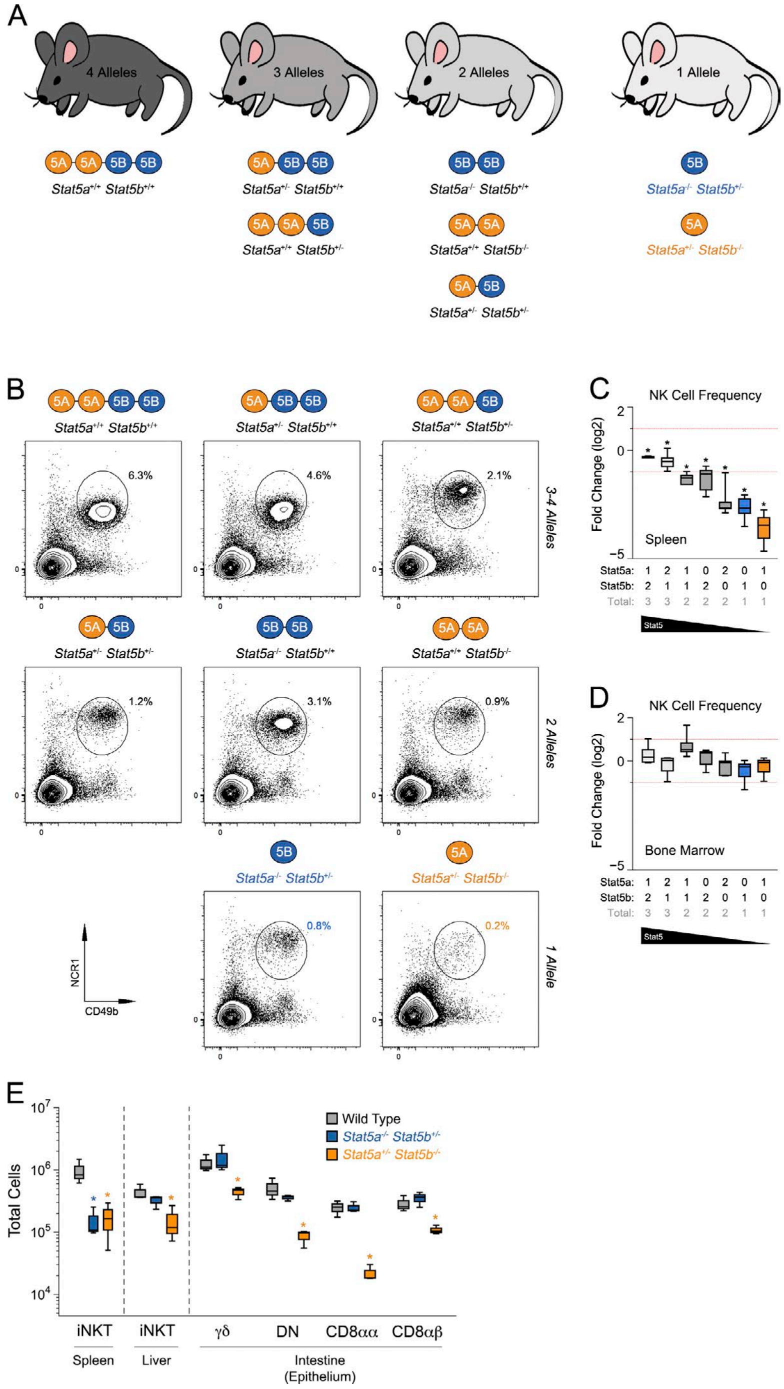

Figure S1. **A genetic model for studying STAT5 function in ILCs and ILTCs.** (A) Cartoon depicts the mouse models used in this study. The eight genotypes are grouped on the basis of the total number of STAT5 alleles, ranging from four (two alleles each of *Stat5a* and *Stat5b*) to one (one allele of either *Stat5a* or *Stat5b*). The rightmost genotypes, *Stat5a*$^{-/-}$ *Stat5b*$^{+/-}$ and *Stat5a*$^{+/-}$ *Stat5b*$^{-/-}$, are referred to as one-allele STAT5A-deficient and one-allele STAT5B-deficient (respectively), indicating that they lack both alleles of one paralog and retain only one of the other paralog. Throughout the article, the former is represented by blue coloring and the latter by orange coloring. (B) Contour plots show percentages of splenic NK cells for each genotype. (C and D) Box plots indicate the frequency of spleen (C) or bone marrow (D) NK cells. Number of *Stat5a*, *Stat5b*, and total alleles is explained in the key below. Data are compiled from five experiments and presented as the $\log_2$ fold change in percentage of NK cells relative to WT controls (WT = 0). Dotted lines denote twofold changes, and asterisks denote significant differences (P < 0.05, Student's *t* test) compared with WT controls. (E) Box plot shows total counts for invariant NKT cells (iNKT), γδ T cells, CD4/CD8 "double-negative" (DN) T cells, CD8αα T cells, and conventional CD8αβ T cells within the indicated tissues. Data are compiled from two to four experiments, depending on tissue and subset ($n \geq 3$ mice/genotype). *, P < 0.05 (Student's *t* test) compared with WT controls.

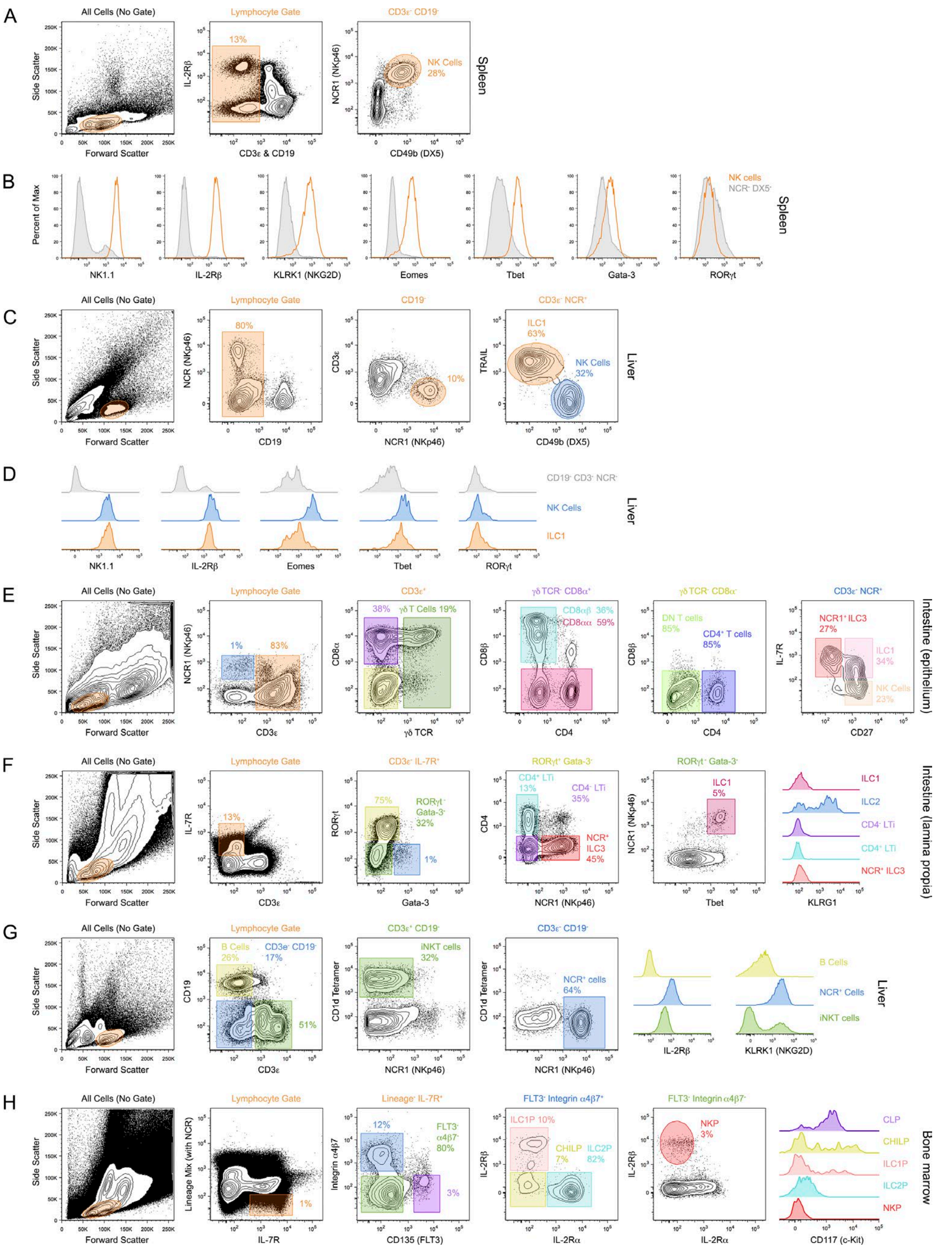

Figure S2. **Gating strategies and phenotype confirmations for ILCs, ILTCs, and progenitors.** (A–H) Dot plots show flow cytometry gating strategies. (A) Spleen and bone marrow NK cells: $CD3\varepsilon^-$ $CD19^-$ $NCR1^+$ $CD49b^+$. (C) Liver ILC1: $CD3\varepsilon^-$ $CD19^-$ $NCR1^+$ $TRAIL^+$ $CD49b^-$, NK cells: $CD3\varepsilon^-$ $CD19^-$ $NCR1^+$ $TRAIL^-$ $CD49b^+$. (E) Intestinal epithelium γδ T cells: $CD3\varepsilon^+$ γδ TCR^+, double-negative (DN) T cells: $CD3\varepsilon^+$ $CD8\beta^-$ $CD8\alpha^-$ $CD4^-$, CD8αα T cells: $CD3\varepsilon^+$ $CD4^-$ $CD8\alpha^+$ $CD8\beta^-$, CD8αβ T cells: $CD3\varepsilon^+$ $CD4^-$ $CD8\alpha^+$ $CD8\beta^+$, ILC1: $CD3\varepsilon^-$ $NCR1^+$ $IL\text{-}7R^{high}$ $CD27^+$, NK cells: $CD3\varepsilon^-$ $NCR1^+$ $IL\text{-}7R^-$ $CD27^+$, $NCR1^+$ ILC3: $CD3\varepsilon^-$ $NCR1^+$ $IL\text{-}7R^{high}$ $CD27^-$. (F) Intestinal lamina propia ILC1: $CD3\varepsilon^-$ $IL\text{-}7R^+$ $ROR\gamma t^-$ $GATA\text{-}3^-$ $NCR1^+$ $T\text{-}BET^+$, ILC2: $CD3\varepsilon^-$ $IL\text{-}7R^+$ $ROR\gamma t^-$ $GATA\text{-}3^+$ (or $KLRG1^{high}$), ILC3 (all subsets): $CD3\varepsilon^-$ $IL\text{-}7R^+$ $ROR\gamma t^+$ $GATA\text{-}3^-$, $NCR1^+$ ILC3: $CD3\varepsilon^-$ $IL\text{-}7R^+$ $ROR\gamma t^+$ $GATA\text{-}3^-$ $NCR1^+$ $CD4^-$, $CD4^+$ LTi,: $CD3\varepsilon^-$ $IL\text{-}7R^+$ $ROR\gamma t^+$ $GATA\text{-}3^-$ $NCR1^-$ $CD4^+$, $CD4^-$ LTi,: $CD3\varepsilon^-$ $IL\text{-}7R^+$ $ROR\gamma t^+$ $GATA\text{-}3^-$ $NCR1^-$ $CD4^-$. (G) Spleen and liver NKT cells: $CD3\varepsilon^+$ $CD19^-$ $NCR1^-$ CD1d-tetramer$^+$. (H) Common lymphoid progenitors (CLPs): lineage$^-$ $IL\text{-}7R^+$ $CD135^+$ Integrin $\alpha4\beta7^-$ $CD117^+$, common progenitors to all helper-like ILCs (CHILPs): lineage$^-$ $IL\text{-}7R^+$ $CD135^-$ Integrin $\alpha4\beta7^+$ $IL\text{-}2R\alpha^-$ $IL\text{-}2R\beta^-$ $CD117^+$, ILC1p: lineage$^-$ $IL\text{-}7R^+$ $CD135^-$ Integrin $\alpha4\beta7^+$ $IL\text{-}2R\alpha^-$ $IL\text{-}2R\beta^+$ $CD117^+$, ILC2p: lineage$^-$ $IL\text{-}7R^+$ $CD135^-$ Integrin $\alpha4\beta7^+$ $IL\text{-}2R\alpha^+$ $IL\text{-}2R\beta^-$ $CD117^-$, NKp: lineage$^-$ $IL\text{-}7R^+$ $CD135^-$ Integrin $\alpha4\beta7^-$ $IL\text{-}2R\alpha^-$ $IL\text{-}2R\beta^+$ $CD117^-$. (B) Histograms confirm that the $CD3\varepsilon^-$ $CD19^-$ $NCR1^+$ $CD49b^+$ population from the spleen contains $NK1.1^{high}$ $IL\text{-}2R\beta^{high}$ $KLRK1^{high}$ $EOMES^{high}$ $T\text{-}BET^{high}$ $GATAT\text{-}3^{low}$ $ROR\gamma t^-$ NK cells. (D) Flow cytometry histograms confirm that $CD3\varepsilon^-$ $CD19^-$ $NCR1^+$ $TRAIL^+$ $CD49b^-$ and $CD3\varepsilon^-$ $CD19^-$ $NCR1^+$ $TRAIL^-$ $CD49b^+$ populations in the liver contain $NK1.1^{high}$ $IL\text{-}2R\beta^{high}$ $EOMES^-$ $T\text{-}BET^{high}$ $ROR\gamma t^-$ ILC1 and $NK1.1^{high}$ $IL\text{-}2R\beta^{high}$ $EOMES^{high}$ $T\text{-}BET^{high}$ $ROR\gamma t^-$ NK cells, respectively.

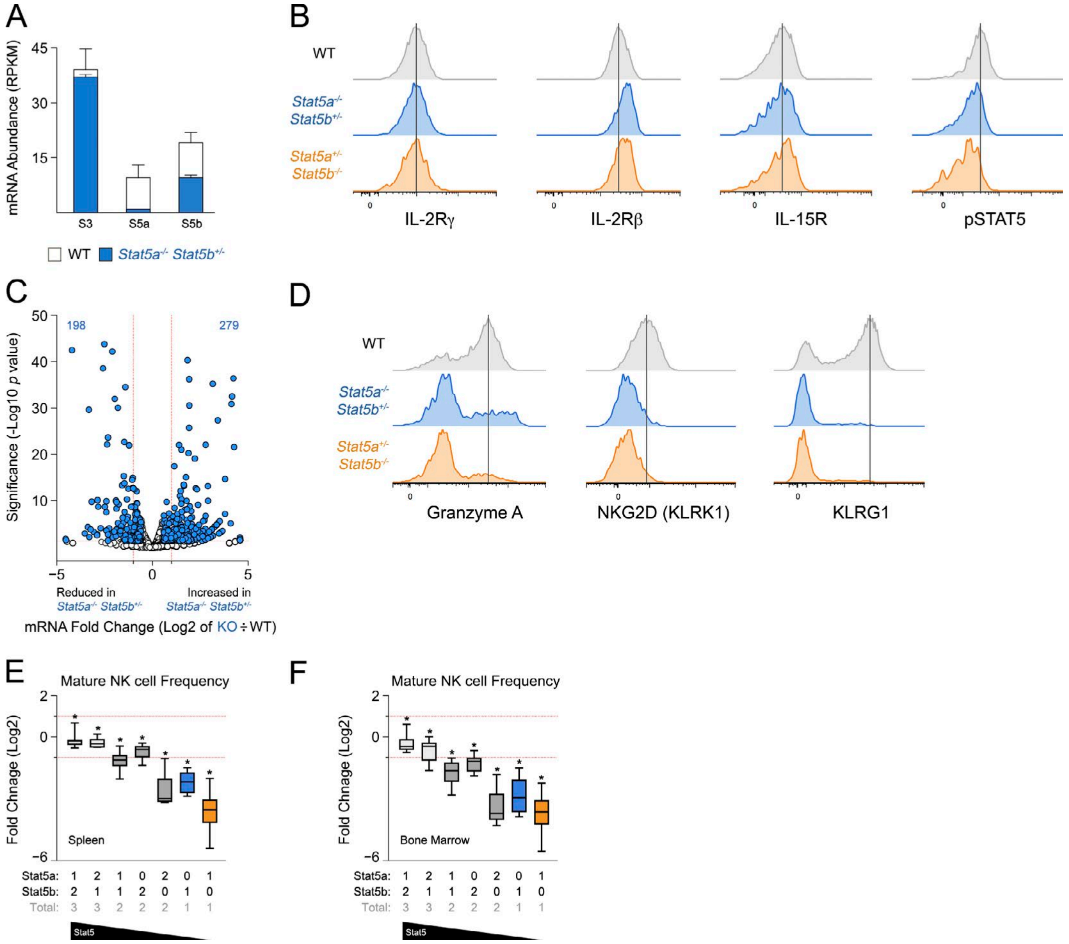

Figure S3. **Transcriptomic and phenotypic analysis of STAT5-deficient NK cells.** (A and C) Transcriptomes were measured in splenic NK cells from WT and one-allele STAT5A-deficient mice (*Stat5a*$^{-/-}$ *Stat5b*$^{+/-}$). Bar graph shows RPKM values for STAT3, STAT5A, and STAT5B transcripts. (B) Flow cytometry histograms show protein levels for IL-15 receptor components on the surface of splenic NK cells. Rightmost histogram shows tyrosine phosphorylation of STAT5 in response to IL-15 (1 h). (C) Volcano plot shows fold change and variance for all transcripts relative to WT controls. Positively (right) and negatively regulated (left) genes are highlighted in blue and summed. Dotted lines indicate twofold changes and p-value of 0.05. (D) Flow cytometry histograms show protein levels for Granzyme A, KLRK1, and KLRG1. (E and F) Box plots indicate the frequency of mature KLRG1^{high} IGAM^{high} NK cells in spleen and bone marrow. Genotypes are explained in the key below. Data are compiled from five experiments and presented as $\log_2$ fold change relative to WT controls (WT = 0). Dotted lines denote twofold changes, and asterisks denote significant differences (*, $P < 0.05$, Student's *t* test) compared with WT controls. (A and C) Two biological replicates were included per genotype. (B and D) Data are representative of three or four experiments ($n \geq 3$ mice/genotype).

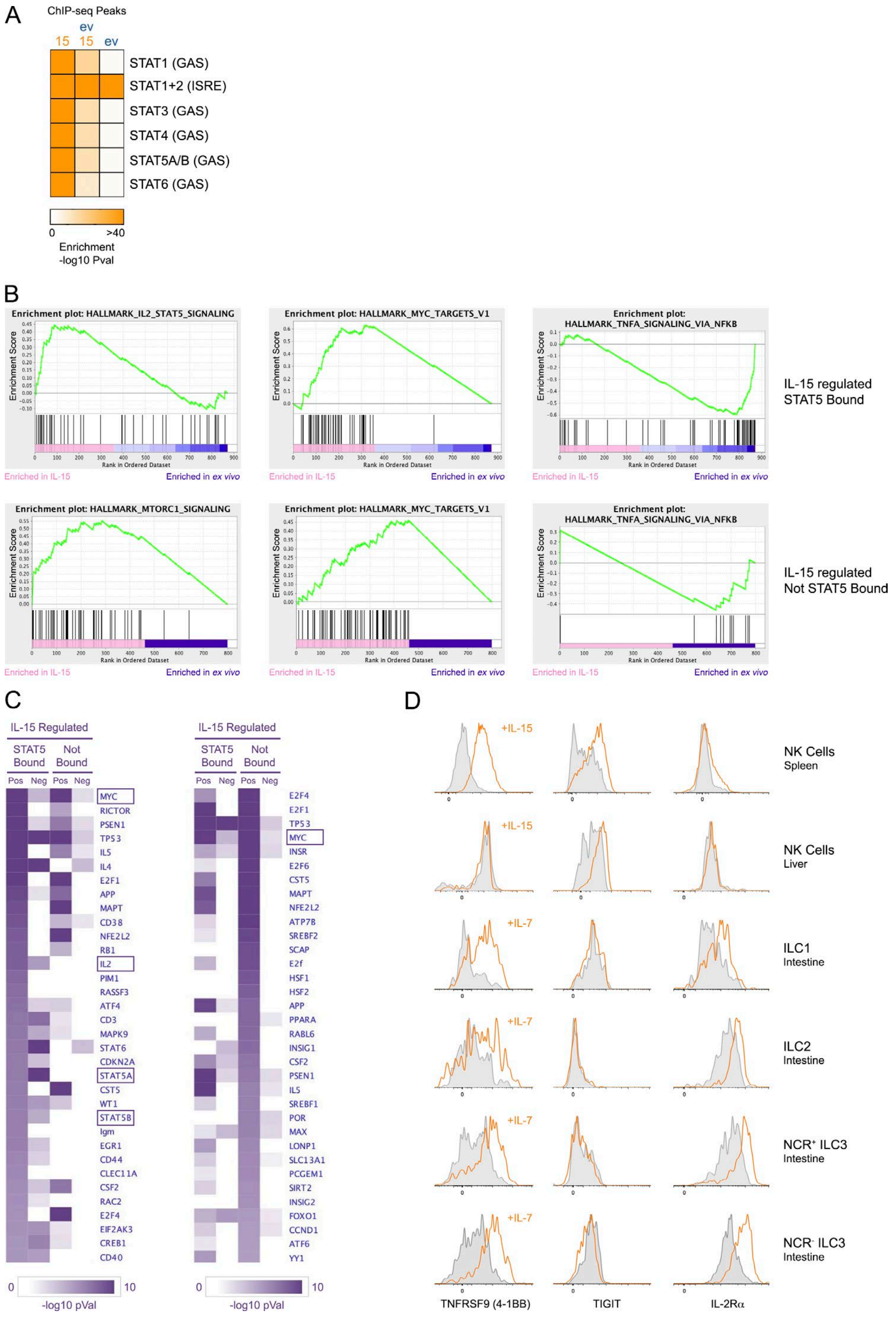

Figure S4. **STAT5-dependent and -independent transcriptional effects downstream of cγ cytokines.** (A) Heat map shows enrichment for STAT-binding motifs under STAT5 peaks detected in ex vivo (abbreviated as "ev" and written in blue) and/or IL-15–treated NK cells (abbreviated as "15" and written in orange). Two biological replicates were included per condition. (B) GSEA curves show enrichment of "hallmark" gene sets in transcriptomes of ex vivo or IL-15–treated NK cells. (C) Heat maps show prediction significance scores (presented as $-\log_{10}$ p-values) for upstream regulators likely to explain transcriptional changes associated with positively (pos) or negatively (neg) regulated STAT5-bound or STAT5-unbound gene sets. Left histogram is sorted in descending order on the basis of STAT5-bound, positively regulated genes. Right histogram sorted in descending order on the basis of STAT5-unbound, positively regulated genes. (B and C) Three biological replicates were included per condition for RNA-seq. (D) Flow cytometry histograms show protein levels for IL-15/STAT5 signature genes TNFRSF9 (4-1BB), TIGIT, and IL-2Rα on the indicated ILC subsets before and after treatment with cγ cytokines (IL-7 or IL-15). Data are representative of two or three experiments ($n \geq 3$ mice/genotype).

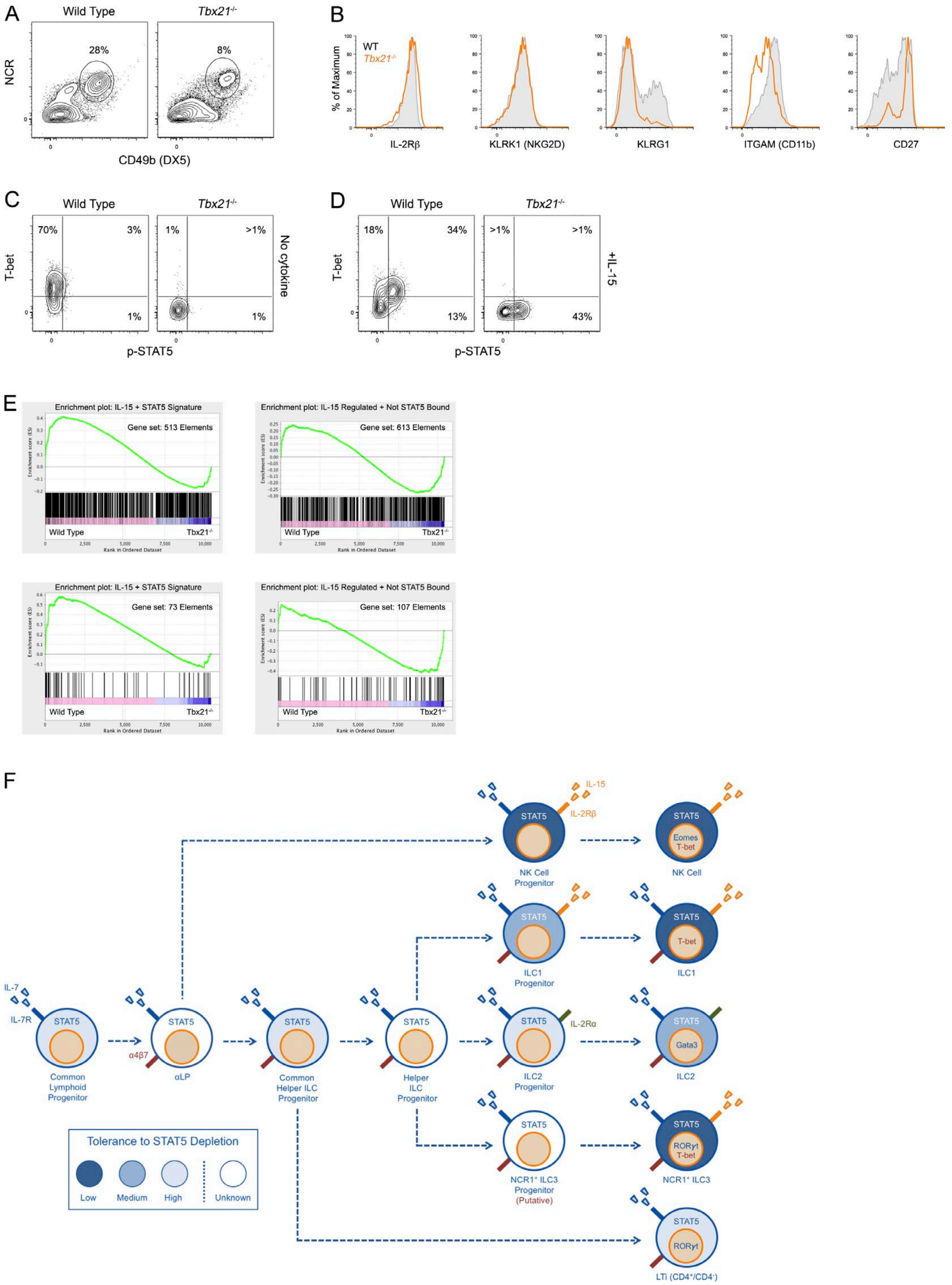

Figure S5. **Evidence for coordinated and cooperative functions of T-BET and STAT5 in NK cells.** (A) Contour plots show percentages of splenic NK cells within the CD3ε⁻ CD19⁻ compartment of WT and T-BET–deficient mice. (B) Histograms show protein levels for IL-2Rβ, KLRK1, KLRG1, and ITGAM on the surface of splenic NK cells. (C and D) Contour plots show percentages of T-BET and/or phospho-STAT5 positive splenic NK cells before and after treatment with IL-15. (A–D) Data are representative of two experiments (n = 2 mice/genotype). (E) Transcriptomes were measured in IL-15–treated splenic NK cells from WT or T-BET–deficient mice. GSEA curves show enrichment of IL-15/STAT5 signature or IL-15–regulated, STAT5-unbound gene sets. Two biological replicates were included per genotype. (F) Cartoon depicts ILC ontogeny and the relative ability of each subset or progenitor to tolerate STAT5 depletion on the basis of cellular phenotypes of one-allele STAT5-deficient mice. Color shading indicates degree of tolerance (i.e., darker colors indicate lower tolerance and greater loss of total cells relative to WT controls). White indicates known or putative progenitors that were not assessed.

Table S1 contains RPKM measurements, fold change calculations, and p-values for RNA-seq experiments. Table S2 contains annotated STAT5 ChIP-seq data. Table S3 contains enrichment ranks and p-values for TF-binding motifs found under STAT5 peaks. Table S4 defines the STAT5 gene signature in NK cells and all gene sets used for transcriptome analysis. Tables S1–S4 are provided as Excel files.
